# Interruption of neutrophil extracellular traps formation dictates host defense and tubular HOXA5 stability to augment efficacy of anti-Fn14 therapy against septic AKI

**DOI:** 10.7150/thno.61902

**Published:** 2021-09-13

**Authors:** Yin Ni, Bang-Chuan Hu, Guo-Hua Wu, Zi-Qiang Shao, Yang Zheng, Run Zhang, Jun Jin, Jun Hong, Xiang-Hong Yang, Ren-Hua Sun, Jin-Quan Liu, Shi-Jing Mo

**Affiliations:** 1 Department of Intensive Care Unit, Zhejiang Provincial People's Hospital, People's Hospital of Hangzhou Medical College, Hangzhou 310014, Zhejiang, P.R. China.; 2 Zhejiang University School of Medicine, Zhejiang University, Hangzhou 310029, Zhejiang, P.R. China.

**Keywords:** Neutrophil extracellular traps, Fibroblast growth factor-inducible molecule 14, Macrophages, Homeobox protein Hox-A5, Septic acute kidney injury

## Abstract

The immunosuppressive, inflammatory microenvironment orchestrated by neutrophil extracellular traps (NETs) plays a principal role in pathogenesis of sepsis. Fibroblast growth factor-inducible molecule 14 (Fn14) has been established as a potential target for septic acute kidney injury (AKI), making further therapeutic benefits from combined NETs and Fn14 blockade possible.

**Methods:** The concurrence of NETs and Fn14 in mice and patients with septic AKI were assessed by immunofluorescence, immunohistochemistry, enzyme-linked immunosorbent assay (ELISA) and *in silico* studies. Survival, histopathological and biochemical analyses of wild-type and PAD4-deficient *CMV-Cre*; PAD4*^fl/fl^* mice with septic AKI were applied to evaluate the efficacy of either pharmacological or genetic NETs interruption in combination with Fn14 blockade. Molecular mechanisms underlying such effects were determined by CRISPR technology, fluorescence-activated cell sorter analysis (FACS), cycloheximide (CHX) pulse-chase, luciferase reporter and chromatin immunoprecipitation (ChIP) assay.

**Results:** NETs formation is concurred with Fn14 upregulation in murine AKI models of abdominal, endotoxemic, multidrug-resistant sepsis as well as in serum samples of patients with septic AKI. Pharmacological or genetic interruption of NETs formation synergizes with ITEM-2, a monoclonal antibody (mAb) of Fn14, to prolong mice survival and provide renal protection against abdominal sepsis, the effects that could be abrogated by elimination of macrophages. Interrupting NETs formation predominantly perpetuates infiltration and survival of efferocytic growth arrest-specific protein 6^+^ (GAS6^+^) macrophages in combination with ITEM-2 therapy and enhances transcription of tubular cell-intrinsic Fn14 in a DNA methyltransferase 3a (DNMT3a)-independent manner through dismantling the proteasomes-mediated turnover of homeobox protein Hox-A5 (HOXA5) upon abdominal sepsis challenge or LPS stimuli. Pharmacological NETs interruption potentiates the anti-septic AKI efficacy of ITEM-2 in murine models of endotoxemic and multidrug-resistant sepsis.

**Conclusion:** Our preclinical data propose that interrupting NETs formation in combination with Fn14 mAb might be a feasible therapeutic strategy for septic AKI.

## Introduction

Septic acute kidney injury (AKI) represents the most common and life-threatening organ failure in intensive care unit (ICU) as a result of deregulation in host defense against severe and persistent infection, which leads to substantial morbidity and lethality 1. Despite considerable advancements in the past decades, therapeutic approaches for patients with septic AKI are limited and rightfully classified as only incremental 2, 3. Hence, there is a pressing need to develop innovative strategy with potential for long-term clinical prognosis and cures for this debilitating disease.

Neutrophils are an important portion of innate immune cells responsible for releasing neutrophil extracellular traps (NETs) to eradicate foreign pathogens during infection 4, 5. However, NETs become pathogenic after persistent perturbation of the pro-/anti-inflammatory homeostasis, constituting the pathophysiological feature of systemic inflammatory response syndrome (SIRS) 6. The NETs-extruded products, including citrullinated histones (Cit-H3), granular enzymes neutrophil elastase (NE) and extracellular DNA, function as the damage‐associated molecular pattern molecules (DAMPs) to initiate immunosuppression 7, which is found to be associated with the extremely rapid course of inflammatory storm and tissue injury elicited by sepsis 8. Emerging evidences support the vital role of NETs formation in exacerbating sepsis and predicting clinical outcomes of patients 9, 10, while blockade of NETs formation enables survival improvement in mice with lethal sepsis 11. Hitherto, the significance of NETs formation in directing host defense against septic AKI is still undefined and it remains unknown whether NETs can be an immune checkpoint for this disease.

Fibroblast growth factor-inducible molecule 14 (Fn14) has been implicated in pathogenesis of chronic inflammatory diseases and AKI 12-14. The pleiotropic transmembrane receptor evokes a series of downstream signalling routes once being activated by the ~27 kDa ligand TNF-like weak inducer of apoptosis (TWEAK), which can result in inflammatory evasion, cell apoptosis and tissue injury. Several studies identify the potential of targeting tubular TWEAK/Fn14 cascade as a plausible approach for AKI treatment. For instance, the TWEAK-deficient mice are refractory to tubular damage and nephrotoxicity caused by cyclosporin A (CsA), which eventually upregulates Fn14 expression in kidney epithelial cells 15. Genetic ablation of Fn14 diminishes proteinuria IgG deposition and repairs glomerular filtration barrier in mice with lupus nephritis 16. Our recent research demonstrated that tubular Fn14 is upregulated during abdominal sepsis and, pharmacological deactivation of tubular cell-intrinsic Fn14 by ITEM-2, an anti-Fn14 monoclonal antibody (mAb), prolongs survival of mice and protects them against septic AKI 17. Systemic delivery of microRNA-19a mimics into cecal ligation and puncture (CLP) and the LPS-induced endotoxemia (LIE) mice downregulates tubular cell-intrinsic Fn14 expression, thereby impairing AKI development 18. These evidences underscore that Fn14 might be a promising therapeutic target for septic AKI.

In the present study, data from three distinct murine models of sepsis, serum samples of clinical patients and bioinformatic studies depict that NETs formation is concurred with Fn14 upregulation during septic AKI. Pharmacological blockade of NETs formation by either neutrophil elastase (NE) inhibitor sivelestat (SIVE) or peptidylarginine deiminase 4 (PAD4) inhibitor Cl-Amidine potentiates the efficacy of ITEM-2 to suppress mortality and AKI of mice with abdominal sepsis. Similar benefits were seen in *CMV-Cre*; PAD4*^fl/fl^* mice receiving ITEM-2 therapy. Eliminating macrophages abrogates the synergistic effects of combined NETs and Fn14 blockade, which may account for the increased infiltration and survival of growth arrest-specific protein 6^+^ (GAS6^+^) macrophages within kidney tissues upon combination therapy. Mechanistical studies reveal that either PAD4 deficiency or SIVE plus Cl-Amidine cotreatment augments transcription of tubular cell-intrinsic Fn14 in a DNA methyltransferase 3a (DNMT3a)-dispensable fashion through dismantling the proteasome-mediated turnover of homeobox protein Hox-A5 (HOXA5) upon abdominal sepsis challenge or LPS stimuli. Combined blockade of NETs and Fn14 synergistically prevents mortality and AKI of mice with LIE and multidrug-resistant sepsis (MDRS). Our findings elucidate a mechanism whereby NETs blockade governs host defense and enhances the effectiveness of Fn14 mAb against septic AKI, thus supporting further clinical investigation of this combination strategy as a rational, therapeutic strategy to favor patients with septic AKI.

## Materials and methods

### Reagents, constructs and antibodies

The *Escherichia coli* 0111: B4 LPS was obtained from Sigma-Aldrich (St. Louis, MO, USA). Multidrug-resistant *Staphylococcus aureus* (MDRSA, BAA-44^TM^) was from the American Type Culture Collection (ATCC) and grown in trypticase soy broth (BD Biosciences) with aeration at 37 °C. The anti-Fn14 monoclonal antibody (mAb) ITEM-2 was as previously described 17. Sivelestat (cas#201677-61-4) was from Cayman Chemical (Ann Arbor, MI). Cl-Amidine (cat#S8141) was purchased from Selleckchem (TX, USA). Anti-CSF1R (AFS98, #BP0213-R025MG) antibody was ordered from BioXCell (West Lebanon, New Hampshire, USA). Clodronate liposome was purchased from www.ClodronateLiposomes.org. Small interfering RNAs (siRNAs) targeting mouse SPIB, HOXA5, RXRA, ESRRB, RORA_1, CREB1 and DNMT3a were ordered from Dharmacon (Lafayette, CO). HOXA5 expression plasmids were constructed by subcloning the open reading frame of HOXA5 cDNA into the multiple cloning sites of pSF-CMV-NH2-FLAG vector (Sigma-Aldrich, St. Louis, MO, USA). Short hairpin RNAs (shRNAs) duplexes targeting HOXA5 and HOXA1 were generated by subcloned respective shRNA oligos into lentiviral pLKO.1-Puro vector (Sigma-Aldrich). Commercial pCRISPR-LvSG03 vector (GeneCopoeia, Rockville, USA) was used to generate the CRISPR/Cas9-mediated PAD4 knockout. The following antibodies with the company and catalogue no. were used for immunohistochemical staining (IHC), immunofluorescence (IF), fluorescence-activated cell sorter analysis (FACS), western-blotting (WB) or chromatin immunoprecipitation (ChIP) analyses: anti-citrullinated-histone H3 (Abcam, #ab5103), anti-NE (Abcam, #ab68672), anti-Fn14 (Santa Cruz Biotechnology, #sc-56250), anti-PAD4 (Abcam, #ab96758), anti-NGAL (Abcam, #ab70287), anti-F4/80 (BioLegend, #123118), anti-Ly6G (BioLegend, #127624), anti-CD45 (BioLegend, #103110), anti-CD68 (Abcam, #ab201340), anti-GAS6 (Santa Cruz Biotechnology, #sc-376087), anti-CD3 (Abcam, #ab5690), anti-CD8 (Abcam, #ab22378), anti-CD4 (Santa Cruz Biotechnology, #sc-19641), anti-FOXP3 (Santa Cruz Biotechnology, #sc-53876), anti-CD115 (Abcam, #ab271294), anti-DR5 (Cell signalling Technology, #8074), anti-RANK (Cell signalling Technology, #4845), anti-HOXA5 (Santa Cruz Biotechnology, #sc-365784), anti-HOXA1 (Santa Cruz Biotechnology, #sc-293257), anti-SPIB (Cell signalling Technology, #14337), anti-RXRA (Cell signalling Technology, #3085), anti-ESRRB (Abcam, #ab19331), anti-RORA (Cell signalling Technology, #16540), anti-CREB1 (Cell signalling Technology, #9197) and anti-GAPDH (Biosynthesis, #bs-0755R).

### Human subjects

Serum samples of 42 healthy volunteers and 93 septic AKI patients were obtained from Intensive Care Unit, Zhejiang Provincial People's Hospital. The baseline characteristics of human subjects with various Sequential Organ Failure Assessment (SOFA) scores and Kidney Disease Improving Global Outcomes (KDIGO) stages are listed in Table S1. The written informed consents were obtained from all human subjects, which were not involved in previous procedures and test naive. Study protocols concerning human subjects are consistent with the principles of the Declaration-of-Helsinki and approved by the Clinical Research Ethics Committee of Zhejiang Provincial People's Hospital, Hangzhou Medical College.

### Mice, septic AKI models and *in vivo* procedures

PAD4*^flox/flox^* mice (B6(Cg)-*Padi4^tm1.2Kmow^*/J, 026708) and CMV-Cre mice (B6.C-Tg(CMV-cre)1Cgn/J, 006054) were purchased from the Jackson Laboratory. PAD4*^flox/flox^* mice were crossed with CMV-Cre mice to generate the PAD4-deficient CMV-*Cre*; PAD4*^fl/fl^* mice. C57BL/6-*Tnfrsf12α^em1Smoc^* mice (Fn14-knockout [KO], # NM-KO-200539) were purchased from Shanghai Biomodel Organism Science and Technology Company (Shanghai, China). All animals were bred in the specific-pathogen-free animal facility of the Animal Experiment Center of Zhejiang University. Septic AKI models of cecal ligation and puncture (CLP) and the LPS-induced endotoxemia (LIE) were performed as described previously 17-20. For CLP, a small midline incision was made in abdomen of mice after being anesthetized with ketamine (80 mg/kg). The cecum was exteriorized without intestinal obstruction and then punctured at a distance 5.0 mm from the cecal tip away from the ileocecal valve. After that, the abdomen was closed in two layers to prevent leakage of fluid. For LIE, mice were intraperitoneally injected with LPS at a dosage of 10 mg/kg for the indicated time points before kidney excision. To establish multidrug-resistant sepsis (MDRS), mice were intraperitoneally infected with 0.1 mL bacterial suspension (5 × 10^8^ colony-forming units [CFUs] per mouse). Mortality was assessed for at least 7 days after the onset of sepsis to ensure that no additional deaths occurred. All animal studies were conducted with the approval of the Zhejiang University Institutional Animal Care and Use Committee and were performed in accordance with established guidelines.

Sivelestat (SIVE) was formulated in dimethyl sulfoxide (DMSO) at a concentration of 15 mg/mL. For *in vivo* administration, mice were received 100 mg SIVE per kg body weight by intraperitoneal injection at the indicated time points before or after sepsis challenge. ITEM-2 was utilized at a concentration of 0.5 mg, a dose with demonstrated efficacy in our previous murine models of septic AKI 17. For therapeutic evaluation, mice were treated with SIVE or ITEM-2 as single agent or in combination until completion of the study. To eliminate macrophages, anti-CSF1R antibody was administered every thirty-six hours (300 μg per mouse intraperitoneally), with administration maintained over the course of the experiment. Clodronate liposome was reconstituted in PBS and administered with 200 μL by intravenous (i.v.) injection to eliminate circulating macrophages. The PAD4 inhibitor Cl-Amidine was dissolved in 1:1 DMSO:PBS stocking solution and administered intraperitoneally at a dose of 50 mg/kg body weight. To examine *in vivo* functions of macrophages in CLP mice, F4/80^+^ macophages (1×10^6^) isolated from the indicated mice were transferred into CLP mice receiving anti-CSF1R (300 μg, i.p.) pretreatment by a single tail-vein injection in combination with or without intraperitoneal ITEM-2 therapy. Kidney samples were excised for hematoxylin&eosin (H&E) and immunohistochemical staining at the end time points of experiment. Blood samples were collected from the retroorbital sinus of anesthetized mice into EDTA-coated vacutainer tubes (BD Biosciences), allowed to clot for two hours at room temperature, and then centrifuged for 15 min at 1500 rpm before analysis. Biochemical detection of serum creatinine (Scr) and blood urea nitrogen (BUN) were performed in the Clinical Pathology Laboratory at Zhejiang University. Commercially available enzyme-linked immunosorbent assay (ELISA) kits were used to measure the activity of HMGB1 (#ST51011, Shino-Test Corporation), TNF (#bsk12002, Biosynthesis), IL-1β (#DLB50, R&D Systems) and IL-6 (#P08505, RayBio^®^).

### Serum NETs and Fn14 measurement

Serum levels of human NETs were measured using the PMN (Neutrophil) Elastase enzyme-linked immunosorbent assays (ELISA) Kit (#BMS269, eBioscience, San Diego, CA, USA) according to the manufacturer's instructions. Serum concentrations of mouse NETs (#Q3UP87) and human Fn14 (#Q9NP84) were determined by the colorimetric, sandwich-based RayBio^®^ ELISA Kits (Shanghai, China) according to the manufacturerʼs guidelines. The fluorescence intensity was quantified by a FlexStation 3 Microplate Reader (Molecular Devices, CA, USA).

### Transmission electron microscopy (TEM)

TEM was carried out to detect mitochondrial morphology of kidney tubular epithelial cells in murine models of CLP, LIE or MDRS. Briefly, kidney specimens were fixed with 0.1 M sodium cacodylate buffer containing 2.5% glutaraldehyde, 4% paraformaldehyde and 0.02% picric acid at pH 7.2, followed by secondary fixation with 1% osmium tetroxide and 1.5% potassium ferrocyanide. After being dehydrated with a graded ethanol series, the specimens were subjected to osmosis, embedding, sectioning and staining. Images were obtained with a Hitachi H7700 electron microscope.

### Histopathological and immunohistochemical staining

Kidney tissues were excised from mice, fixed in 10% formalin, decalcified in 10% EDTA and embedded in paraffin for hematoxylin&eosin (H&E) and immunohistochemical staining as described in previous publications 17, 18, 20. For immunohistochemical staining, renal sections were rehydrated and subjected to the heat-mediated antigen retrieval using citrate buffer (pH 6.0). Endogenous peroxidases were blocked with 3% H_2_O_2_ in TBST, and slides were blocked with 5% bovine serum albumin (BSA) in TBST. Sections were probed with the primary antibodies against neutrophil gelatinase-associated lipocalin (NGAL) and diluted in BSA overnight at 4 °C, followed by washing three times with TBST and incubating with horseradish peroxidase-conjugated secondary antibodies diluted in BSA for 1 h at room temperature. Slides were then washed again and signal was attained by Dako ChemMateTM Envision^TM^ Detetcion Kit (DaKo, Glostrup, Denmark) for 5 min at room temperature. Slides were washed with ddH_2_O to stop the reaction and then stained with Harris Hematoxylin for 1 min and rinsed with warm tap water for 5 min. Slides were dipped eight times by 0.25% HCl in 70% ethanol and rinsed with tap water again for 5 min. Images were obtained using an AxioVision Rel.4.6 computerized image-analysis system (Carl Zeiss).

### Immunofluorescence

#### Immunohistofluorescence

Immunohistofluorescence was adopted to identify NETs formation, PAD4 and Fn14 colocalization, CD68^+^/GAS6^+^ macrophages, CD4^+^/FOXP3^+^ regulatory T cells (Treg), CD3^+^ total or CD8^+^ cytotoxic T cells as well as CD115^+^ monocytes in kidney tissues of mice with septic AKI. The five-micrometer-thick, paraffin-embedded and 4% paraformaldehyde (PFA) inflation-fixed murine renal sections were prepared and mounted on glass slides. After dewaxing and rehydrating, sections were permeabilized with 0.1% Triton X-100 for 15 min and blocked with PBS containing 1% BSA and 0.1% Tween-20. The sections were then incubated with the primary antibodies against anti-citrullinated-histone H3 (1:250), anti-NE (1:250), anti-PAD4 (1:100), anti-Fn14 (1:100), anti-CD68 (1:50), anti-GAS6 (1:50), anti-CD4 (1:50), anti-FOXP3 (1:50), anti-CD3 (1:50), anti-CD8 (1:50) and anti-CD115 (1:50) overnight, respectively, followed by detection with Alexa Fluor^®^ 488 (1:500; Abcam) or Alexa Fluor^®^ 647 (1:500; Abcam) secondary antibodies for 1 h at room temperature. Sections were stained with 4', 6-diamidino-2-phenylindole (DAPI) and imaged on a Carl Zeiss (Oberkochen, Germany) Axioimager Z1 microscope. The analyses of immunohistofluorescence staining, cell number and colocalization studies were performed using TissueQuest analysis software (TissueGnostics).

#### Immunocytofluorescence

Immunocytofluorescence was performed as previously described 20, 21. In brief, cells in six-well plates were fixed with 4% formaldehyde in PBS for 15 min at 37 °C. After permeabilizing with 1% Triton X-100 for 10 min, they were blocked with 5% BSA in PBS and Tween-20 (PBST) at 37 °C for 1 h and incubated with the indicated primary antibodies. Twenty-four hours later, cell cultures were incubated with secondary antibody conjugated with Alexa Fluor^®^ 488 or Alexa Fluor^®^ 647 at 37 °C for 1 h. After being washed with 1×PBS for three times, cells were stained by 5 µg/mL DAPI in PBS for 15 min. Immunofluorescence images were captured on a fluorescent microscope (IX71; Olympus, Japan).

### Cell culture

Human kidney proximal tubular epithelial (HK-2) cells were grown in RPMI-1640 (GIBCO, Carlsbad, CA) with 10% heat-inactivated FBS, 2 mM glutamine, 100 U/mL penicillin, and 100 g/mL streptomycin in a humidified incubator with 5% CO_2_ at 37 °C as previously described 17, 18, 20. Renal proximal tubular cells (RPTCs) were isolated from the indicated mice using a previously described procedure with minor modifications 19. In brief, kidney cortices were dissected from the medulla, sliced, minced and filtered through a 70 μm strainer and washed with REBM BulletKit^TM^ medium (Lonza). The samples were centrifuged at 1000 rpm for 5 min to pellet the tubules. The pellets were resuspended in medium and the tubules were placed in collagen-coated plates at 37 °C in 5% CO_2_. Twenty-four hours later, medium containing the tubules were collected and centrifuged at 1000 rpm for 5 min, and then resuspended, transferred to another fresh collagen-coated dish and cultured in a humidified incubator with 95% air and 5% CO_2_.

### Transfection, lentiviral production and CRISPR-Cas9 gene editing

Expression constructs and siRNAs were transfected into tubular cells using Lipofectamine 3000 transfection reagents (Invitrogen, Carlsbad, CA) by following the manufacturer's protocol as previously described 20, 21. Lentivirus carrying pLKO.1 plasmid was produced by co-transfecting HEK293T cells with pRSV-Rev, pMD2.G, pMDLg/pRRE, and pLKO.1-puro shRNA. Viral supernatant was harvested after 72 h by passing through a 0.45 μm filter, and then used to infect tubular cells with the addition of 8 μg/mL polybrene for 24 h. Infected cells were selected with puromycin at 1 μg/mL for 7 days before being used for subsequent analyses. For CRISPR-Cas9-mediated PAD4 gene knockout, tubular cells were transfected with pCRISPR-LvSG03 plasmid carrying PAD4 gRNA and selected with 1 μg/mL puromycin for forty-eight hours. Cells were then transferred into a fresh, complete medium and allowed to form colonies from single cell. Colonies were then picked and expanded for knockout validation by real-time quantitative PCR (RT-qPCR) and western-blotting.

### Fluorescence-activated cell sorter analysis (FACS)

For FACS, renal single cells were prepared using the Mouse Multi Tissue Dissociation Kit (Miltenyi Biotec, #130-110-201) following a standard protocol. Digested kidney tissues were passed through a 40 μm cell strainer into RPMI-1640 medium and were centrifuged at 1000 rpm for 5 min at 4 °C. Erythrocytes were eradicated by hypotonic lysis for 1 min at room temperature and renal single cell suspensions were stained with Ghost Dye^TM^ Violet 450 for 15 min in dark and then with indicated antibodies for 30 min on ice. FACS was performed on a CyAn ADP Analyzer (Beckman Coulter) and data were analyzed using FlowJo software (Tree Star, Ashland, OR). To detetct calcein-acetoxymethyl ester (AM) release, the sorted macrophages were stained with 5 μM calcein-AM (APExBIO, #B7755) in PBS for 1 min at room temperature before flow cytometry.

### Cell viability assay

Trypan blue assay was performed to measure cell viability according to the manufacturer's instructions as previously described 22, 23. Briefly, cells were plated in 6-well plates at a density of 1×10^5^ cells per well. At the indicated end time points of experiment, cells were trypsinized and suspended in 1 mL PBS. The cell suspension were then mixed with 100 μL trypan blue solution (Sigma-Aldrich) and the number of viable cells was measured using the Bio-Rad automated cell counter.

### Protein extraction and western blotting

Protein extraction and western blotting analyses were carried out as described in previous publications 17, 18, 20-22. For protein isolation in tissues, septic mice receiving various therapies were sacrificed 1 h before kidneys harvested and snap frozen in liquid nitrogen at -80 °C. Samples were lysed in NP-40 lysis buffer containing 0.5% NP-40, 50 mM Tris-HCl (PH7.4), 50 mM NaCl and protease inhibitor cocktail. Protein lysates were quantified by the Pierce BCA Protein Assay Kit (Life Technologies) and subjected to 10% sodium dodecyl sulfate polyacrylimide gel electrophoresis (SDS-PAGE) for western-blotting. For protein extraction in tubular cells, cells washed twice with cold PBS were solubilized on ice in radioimmunoprecipitation assay (RIPA) buffer (Cwbiotech, Beijing, China). After denaturation, equal amounts of proteins were resolved on 10% SDS-PAGE, transferred to the Immobilon™ PVDF Transfer Membranes (Millipore Corporation, Billerica, MA), blocked with TBST containing 5% BSA and then incubated with the indicated primary antibodies, followed by HRP-linked secondary antibodies incubation for 1h. Bands were visualized by western chemiluminscent HRP substrate kit (PPLYGEN, Beijing, China) and quantified in the dynamic range using the Gel analysis module in ImageJ software.

### Cycloheximide (CHX) pulse-chase experiment

CHX pulse-chase assay was performed as described before 17, 20. Briefly, cells were seeded on 6-well plate at a density of 1×10^6^ cells per well. After culturing overnight, cells were underwent various treatments as desired. Forty-eight hours later, cells were treated with 20 µg/mL CHX dissolved in DMSO. Protein lysates were collected at the indicated time points and subjected to western-blotting analyses for HOXA5 turnover and protein loading controls.

### Real-time quantitative PCR (RT-qPCR)

The procedures of RNA extraction and RT-qPCR were described previously 18, 20, 22, 23. In brief, cells in culture were lysed in Trizol (Life technology, CA) following medium removal. RNA extraction was performed according to the manufacturer's instructions. The extracted RNA was reversely transcribed into cDNA using PrimeScript^®^ RT Reagent Kit (Takana, Dalian, China) with the following modifications: 2 μg of RNA samples with the addition of primers were first denatured at 70 °C for 5 min and cooled down on ice before the addition of buffer and reverse transcriptase. The cDNA samples were diluted and used for PCR amplification on an Applied Biosystems 7900HT cycler with Takana SYBR^®^ Primix Ex Taq*™* Kit (Takana, China) and gene specific primers as the follows:

- Fn14 sense, 5′-GTGTTGGGATTCGGCTTGGT-3′ and Fn14 antisense, 5′-GTCCATGCACTTGTCGAGGTC-3′;
- DR5 sense, 5′-CGGGCAGATCACTACACCC-3′ and DR5 antisense, 5′- AGTTCCCTTCTGACAGGTACTG-3′;
- RANK sense, 5′-CCAGGAGAGGCATTATGAGCA-3′ and RANK antisense, 5′-ACTGTCGGAGGTAGGAGTGC-3′;
- GAPDH sense, 5′-AGGTCGGTGTGAACGGATTTG-3′ and GAPDH antisense, 5′-GGGGTCGTTGATGGCAACA-3′.

Data were normalized to the geometric mean of housekeeping gene glyceraldehyde 3-phosphate dehydrogenase (GAPDH) to control the variability in expression levels and calculated as 2^-[(*C*t of gene) - (*C*t of GAPDH)]^, where *C*t represents the threshold cycle for each transcript normalized to GAPDH and presented as fold changes of gene expression in the test sample compared to the control.

### Luciferase assays

The full-length Fn14 gene promoter sequence containing HOXA5 binding site were cloned into pGL3 luciferase reporter plasmid (Promega, Madison, WI) with the restricted enzyme sites *SacI* and *XmaI*. Dual-luciferase reporter (DLR) assays were determined by Dual Luciferase Reporter Assay Kit (Promega, USA) according to the manufacturerʼs recommendation as reported previously 21-23. In brief, cells plated at a density of 1×10^4^ cells per well in 96-well plates were transfected with 100 ng of Fn14 promoter luciferase reporter plasmids and 1 ng of pRL-TK Renilla plasmid using Lipofectamine 3000 reagent. Forty-eight hours after transfection, cells were harvested and their luciferase activities were measured. Results were presented as averages of at least three independent experiments and normalized for transfection efficiency using Renilla luciferase. The primers used for DLR in the current study were as following:

5′-CGAGCTATCTCCACCCCAGAATTGCAA-3′ (forward) and 5′-CCCCGGGGTGCAAACTTGAGTACCCACG-3′ (reverse).

### Chromatin immunoprecipitation (ChIP)

ChIP was performed in RPTCs and HK-2 cells as described previously according to the manufacturer's protocol (Pierce Agarose ChIP Kit, Thermo) 21-23. In brief, a total of 2×10^6^ cells were crosslinked with 1% formaldehyde at room temperature for 10 min. Cells were incubated with 0.125 M glycine to terminate crosslinking, washed twice with PBS, and re-suspended in the cell lysis buffer (50 mM Tris, 1% NP40, 150 mM NaCl, 0.01% SDS, 1.2 mM EDTA, 1 mM PMSF) for 10 min on ice. Sonicated lysates were diluted in ChIP dilution buffer and immunoprecipitated with 2 μg anti-HOXA5 antibody, 2 μg anti-HOXA1 antibody or 2 μg anti-IgG antibody as a negative control overnight at 4 °C with gentle rocking. Protein/DNA conjugates were eluted from the beads complexes using Elution buffer encompassing 100 mM NaHCO_3_ and 1% SDS for 30 min. Crosslinks were reversed in 300 mM NaCl after RNA and protein were removed by incubation with RNase (Qiagen) at 37 °C for 30 min and proteinase K (Roche) at 45 °C for 1 h. DNA was recovered by phenol/chloroform extraction and ethanol precipitation. The purified DNA fragments were amplified by real-time quantitative PCR with Takana SYBR^®^ Primix Ex Taq^TM^ Kit (Takana, China) with the following primers:

- Fn14-forward (promoter): 5′-TGGAAAGATGGCTCATGGGT-3′ and Fn14-reverse (promoter): 5′-CAACGGATCTCCTGGCAATC-3′;
- Fn14-forward (non-promoter): 5′-GCGTTTCCCAAAGTTCCCTT-3′ and Fn14- reverse (non-promoter): 5′-GAGAAACTGGGACTGGGGAA-3′.

### Bioinformatics

Gene expression data of the GSE60088 and GSE26378 data sets were downloaded from NCBI's Gene Expression Omnibus (GEO). Expression values were Log2 transformed for further Pearson's correlation analysis. The functional association of PAD4 with components of Fn14 signalling cascade was analyzed using protein interaction data from STRING database for generating a set of functional interaction networks.

### Quantification and statistical analyses

Values were presented as mean ± s.d. of at least three independent experiments. SPSS 20.0 software package was used for statistical analyses. Survival was analyzed with the log-rank test. Experiments were repeated at least three times unless otherwise stated. A two-sided *t* test was used for comparisons of two groups; one-way ANOVA with Tukey's post hoc test was used for all other variables. *P* values < 0.05 were considered statistically significant.

## Results

### Concurrence of NETs formation and Fn14 upregulation in septic AKI

We recently employed CLP to establish abdominal sepsis and AKI models in C57BL/6 mice 17, 18, 20. To further validate and extend these findings, septic AKI was evaluated by histopathological examination with hematoxylin-eosin (H&E) staining, by mitochondrial morphology of kidney tubular epithelial cells with transmission electron microscopy (TEM) analyses as well as by serum creatinine (Scr) and blood urea nitrogen (BUN) levels with biochemical detection. H&E staining showed that renal sections from control mice were basically normal, while those from CLP mice exhibited apparent evidences of tubular damage (*P* < 0.001, Figure S1A & B), such as loss of the epithelial brush border, tubular epithelial vacuolization and epithelial desquamation. In line with previous concepts 24, we observed swollen and electron-dense mitochondria in kidney tubular epithelial cells from CLP mice under TEM (Figure S1C). The CLP mice also had increased levels of Scr (*P* < 0.001, Figure S1D) and BUN (*P* < 0.001, Figure S1E) compared with the control mice. These results suggest that murine models of septic AKI elicited by CLP were successfully established.

**Figure 1:**
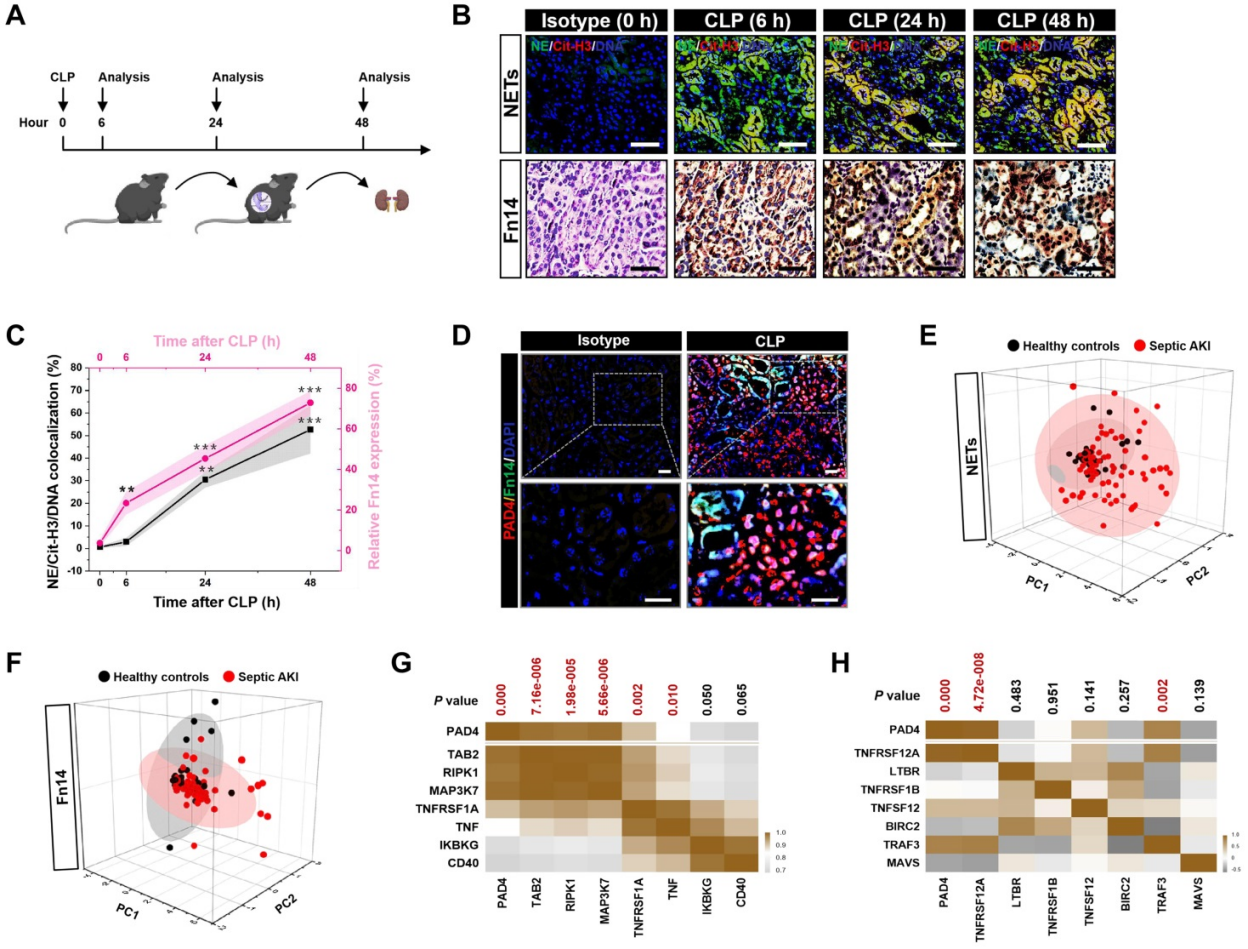
** Concurrence of NETs formation and Fn14 upregulation in septic AKI. (A)** Experimental scheme of immunohistofluorescence and immunohistochemistry analyses for NETs formation and Fn14 expression in mice at the indicated time points after CLP challenge. **(B and C)** Representative pictures (B) and quantification (C) of immunohistofluorescence and immunohistochemistry analyses comparing NETs formation and Fn14 expression in renal sections from CLP mice at the indicated time points. Data are expressed as mean ± s.d. **P < 0.01 and ***P < 0.001 versus 0 h, one-way ANOVA, post hoc comparisons, Tukey's test. Scale bar: 50 µm. **(D)** Representative pictures of PAD4 and Fn14 co-immunofluorescence staining in renal sections from isotype control and CLP mice. Scale bar: 50 µm. **(E and F)** Principal component analysis (PCA) of serum NETs (E) and Fn14 (F) levels in 42 healthy volunteers and 93 patients diagnosed with septic AKI. **(G and H)** Correlation of PAD4 mRNA expression with components of Fn14 signalling cascade in two independent GSE cohorts (GSE60088 [G] and GSE26378 [H]) of sepsis from NCBI's Gene Expression Omnibus (GEO) based on microarray gene expression data. Pearson correlation coefficient was used to calculate the* P* value.

To assess NETs alterations in pathogenesis of septic AKI, kidney samples of mice were collected at 6, 24 and 48 hours after CLP challenge (Figure 1A), respectively. Examination of these samples using immunofluorescence (IF) staining identified a remarkable NETs formation after CLP, as reflected by the higher proportion of neutrophilelastase (NE), citrullinated-histone H3 (Cit-H3) and DNA co-localization in renal sections from CLP mice at 24 h and 48 h than those in renal sections from the isotype control mice, despite such co-localization was implicit at 6 h (Figure 1B). Along with the dynamic NETs formation over times, immunochemistry (IHC) showed that Fn14 protein abundance, which were absent under normal physiological conditions, gradually became detectable at 6 h while markedly elevated at 24 h and peaked at 48 h after CLP challenge (*P* < 0.01 and *P* < 0.001, Figure 1B & C). These data indicate that NETs formation might be concurred with Fn14 upregulation in septic AKI.

The concurrence of NETs formation with Fn14 upregulation in septic AKI was further corroborated by co-immunofluorescence microscopy and western-blotting (WB) analyses showing that the steady-state levels of peptidylarginine deiminase 4 (PAD4), an arginine-citrullines conversion enzyme responsible for NETs release 25, were elevated in renal tissues of CLP mice together with the increased Fn14 expression per se (Figure 1D and S1F). To determine whether the concurrence of NETs formation with Fn14 upregulation is a common feature in septic AKI, we investigated two other clinically relevant mice models: the LPS-induced endotoxemia (LIE) and the multidrug-resistant sepsis (MDRS). Induction of septic AKI in the two models were also confirmed by histopathology, mitochondrial morphology, Scr and BUN levels (*P* < 0.05 and* P* < 0.001, Figure S1G-P), respectively. When compared with the isotype control mice, renal sections of LIE mice displayed remarkable NETs formation in concert with higher levels of Fn14 protein expression (*P* < 0.001, Figure S2A-C). Similar to the observations in CLP and LIE models, mice developed AKI at 48 h after intraperitoneal injection of multidrug-resistant *Staphylococcus aureus* (MDRSA). Their renal sections manifested robust NETs formation to an extent corresponding to the increased accumulation of Fn14 protein (*P* < 0.01 and* P* < 0.001, Figure S2D-F).

To explore the translation of our animal findings to human pathology, we compared serum NETs and Fn14 levels in a cohort of 42 healthy volunteers and 93 clinical patients diagnosed with septic AKI (Table S1). Taking advantage of the linear dimension reduction algorithm with unsupervised principal components analysis (PCA), we found that patients with septic AKI had broader distribution in the up and right quadrants of the graph, while the healthy controls were mainly clustered in the down and left quadrants of the graph (Figure 1E & F), confirming the higher levels of NETs and Fn14 in patients with septic AKI than the healthy controls (Figure S2G & H). When analyzing the relationship between NETs and Fn14 by bioinformatic databases, we found that PAD4 functionally coexpressed with Fn14 signalling network (Figure S2I). Notably and in accordance with these findings, statistically significant correlations between PAD4 and components of Fn14 signalling were observed in two independent GSE cohorts of sepsis from NCBI's Gene Expression Omnibus (GEO) website, which revealed that PAD4 expression was positively correlated with the levels of TGF-beta-activated kinase 1 and MAP3K7-binding protein 2 (TAB2) (*P* = 7.16e-006), receptor-interacting serine/threonine-protein kinase 1 (RIPK1) (*P* = 1.98e-005), mitogen-activated protein kinase kinase kinase 7 (MAP3K7) (*P* = 5.66e-006), tumor necrosis factor receptor superfamily member 1A (TNFRSF1A) (*P* = 0.002) and tumor necrosis factor (TNF) (*P* = 0.010) in GSE60088 (Figure 1G) and with the levels of Fn14 (TNFRSF12A) (*P* = 4.72e-008) and TNF receptor-associated factor 3 (TRAF3) (*P* = 0.002) in GSE26378, respectively (Figure 1H). These data not only reinforce our observations in the murine models and human specimens but also suggest that NETs formation is concurred with Fn14 upregulation during septic AKI.

### Pharmacological blockade of NETs synergizes with Fn14 mAb to confer protection against AKI elicited by abdominal sepsis dependent of macrophages

**Figure 2:**
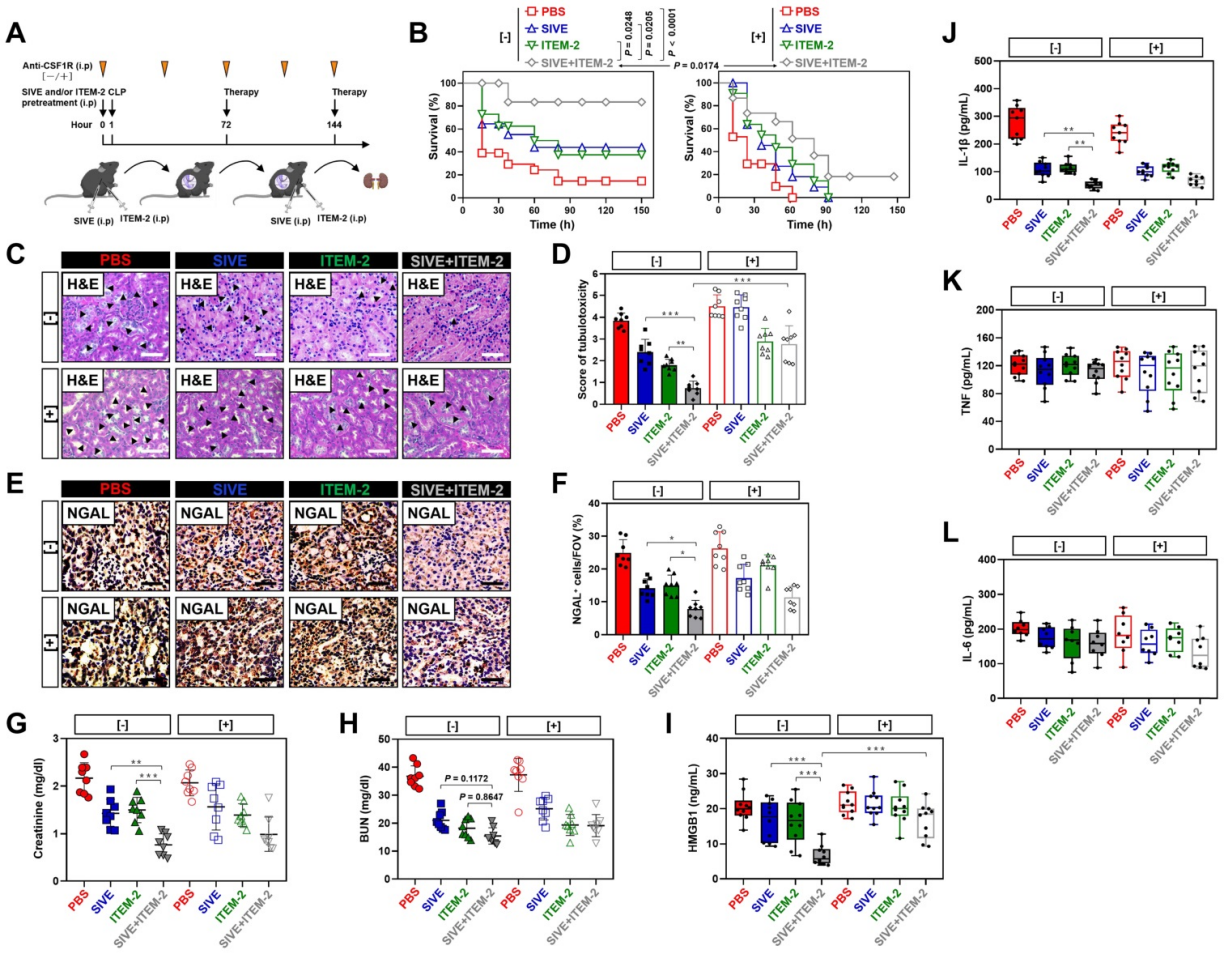
** Pharmacological blockade of NETs synergizes with Fn14 mAb to confer protection against AKI elicited by abdominal sepsis dependent of macrophages. (A)** Experimental scheme of SIVE (100 mg/kg) or ITEM-2 (0.5 mg) monotherapy and SIVE plus ITEM-2 combination therapy for CLP mice with or without intraperitoneal anti-CSF1R (300 µg) pretreatment [-/+] at the indicated time points. **(B)** Kaplan-Meier curves analyzing survivals of mice with intraperitoneal administration of SIVE, ITEM-2 or both in the absence [-] or presence [+] of anti-CSF1R pretreatment at the indicated times after CLP challenge. Log-rank t test was used to caculate the *P* value. **(C and D)** Representative pictures (C) and quantification (D) of H&E staining in renal sections from CLP mice with intraperitoneal administration of SIVE, ITEM-2 or both in the absence [-] or presence [+] of anti-CSF1R pretreatment. Data are expressed as mean ± s.d. **P < 0.01 and ***P < 0.001, one-way ANOVA, post hoc comparisons, Tukey's test. Scale bar: 50 µm. **(E and F)** Representative pictures (E) and quantification (F) of NGAL staining in renal sections from CLP mice with intraperitoneal administration of SIVE, ITEM-2 or both in the absence [-] or presence [+] of anti-CSF1R pretreatment. Data are expressed as mean ± s.d. *P < 0.05, one-way ANOVA, post hoc comparisons, Tukey's test. Scale bar: 50 µm. **(G and H)** Serum creatinine (Scr, G) and blood urea nitrogen (BUN, H) levels in CLP mice with intraperitoneal administration of SIVE, ITEM-2 or both in the absence [-] or presence [+] of anti-CSF1R pretreatment. Data are expressed as mean ± s.d. **P < 0.01 and ***P < 0.001, one-way ANOVA, post hoc comparisons, Tukey's test. **(I-L)** ELISA assays determining serum concentration of HMGB1 (I), IL-1β (J), TNF (K) and IL-6 (L) in CLP mice with intraperitoneal administration of SIVE, ITEM-2 or both in the absence [-] or presence [+] of anti-CSF1R pretreatment. Data are expressed as mean ± s.d. **P < 0.01 and ***P < 0.001, one-way ANOVA, post hoc comparisons, Tukey's test.

Our recent study demonstrated that deactivation of tubular cell-intrinsic Fn14 using a mononal antibody (mAb) ITEM-2 protects mice against the CLP-related AKI 17. Based on the aformetioned data showing that NETs formation was concurred with Fn14 upregulation during septic AKI, we hypothesized that NETs blockade would reprogramme the immunosuppressive, inflammatory microenvironment of CLP mice and then enhance the efficacy of Fn14 mAb therapy. To approach this, mice were injected intraperitoneally with sivelestat (SIVE, a competitive neutrophil elastase [NE] inhibitor, 100 mg/kg, bid), ITEM-2 (0.5 mg, qd) or both for 3-day intervals before CLP challenge at 1 h (Figure 2A, [-]). Mice receiving SIVE injection showed reduced serum NETs levels regardless of ITEM-2 combination, thus confirming eradication of NETs in this model (Figure S3A). As assessed by Kaplan-Meier curves and shown in Figure 2B, a significant improvement of survival duration was observed in response to the combination therapy of SIVE and ITEM-2 (*P* < 0.0001) compared with SIVE (*P* = 0.0205) or ITEM-2 (*P* = 0.0248) therapy alone. Combined SIVE plus ITEM-2 therapy more potently attenuated tubular damage with respect to septic AKI than SIVE or ITEM-2 monotherapy did in the hematoxylin & eosin (H&E) staining (*P* < 0.01 and* P* < 0.001, Figure 2C & D). Immunohistochemical examination of renal sections identified a dramatical reduction in percentage of cells positive for neutrophil gelatinase-associated lipocalin (NGAL), a biomarker of AKI, from CLP mice receiving SIVE plus ITEM-2 combination therapy in contrast to those from mice receiving single therapy (*P* < 0.05, Figure 2E & F). Although either SIVE or ITEM-2 monotherapy allowed CLP mice to increase clearance of Scr, combined therapy did so to a much stronger degree (*P* < 0.01 and* P* < 0.001, Figure 2G). CLP mice receiving combination therapy also had a trend of increased BUN clearance in contrast to those receiving single-agent therapy, albeit such trend did not reach statistical significance (*P* = 0.1172 and *P* = 0.8647, Figure 2H).

As a complementary approach to evaluate the anti-septic AKI effects of combined NETs and Fn14 blockade, release of proinflammatory cytokines including high mobility group box 1 (HMGB1), interleukin-1β (IL-1β), tumor necrosis factor (TNF) and interleukin-6 (IL-6) were measured in CLP mice receiving both SIVE and ITEM-2 therapy. In comparison with monotherapy, combined SIVE and ITEM-2 therapy appreciably reduced serum HMGB1 (*P* < 0.001, Figure 2I) and IL-1β levels (*P* < 0.01, Figure 2J) without significant alterations of TNF (Figure 2K) or IL-6 (Figure 2L) levels. Together, these findings suggest that combined blockade of NETs and Fn14 inhibits release of proinflammatory cytokines that contribute to septic AKI.

To interrogate that the anti-septic AKI phenotypes of combined SIVE plus ITEM-2 therapy are not off-target effects of these agents, we tested whether targeting NETs formation by Cl-Amidine, the peptidylarginine deiminase 4 (PAD4) inhibitor, could enhance the anti-septic AKI efficacy of ITEM-2, equivalent to the findings observed with SIVE. To this end, mice were intraperitoneally administered Cl-Amidine (50 mg/kg, three out of seven days) 1 h prior to CLP challenge with or without ITEM-2 combination. Single Cl-Amidine treatment had minimal effects on rescuing mortality (*P* = 0.1359, Figure S4A) and showed limited ability toward alleviation of HMGB1 despite it abolished NETs formation as efficiently as SIVE treatment did (Figure S4F). However, significant survival improvements were observed in mice receiving combined Cl-Amidine and ITEM-2 treatment (*P* = 0.0043, Figure S4A). Consistently, the tubular damage (*P* < 0.001), Scr (*P* < 0.05), BUN (*P* < 0.01) and HMGB1 (*P* < 0.05) levels in the Cl-Amidine plus ITEM-2-cotreated mice were strikingly lower than those in the Cl-Amidine- or ITEM-2-treated mice (Figure S4B-F). To explore whether endogenous Fn14 signalling in tubular cells is critical for the anti-septic AKI efficacy of NETs blockade, we administered SIVE or Cl-Amidine to Fn14-knockout (KO) mice subjected to CLP, and their survival rate and tubulotoxicity were monitored. In contrast to KO mice receiving no therapy, Fn14-KO mice receiving either SIVE or Cl-Amidine had significantly prolonged survival duration (Figure S4G), and their tubules underwent less injury (*P* < 0.05 and *P* < 0.01, Figure S4H & I). These results suggest that PAD4 inhibitor in combination with Fn14 mAb renders the most powerful ability to counteract septic AKI.

NETs are known to trigger macrophages pyroptosis, which restricts their survival when overactivated 26, 27. We postulated that NETs blockade would be effective in restoring macrophages activity and function so as to direct the anti-septic AKI phenotypes of combination therapy* in vivo* and that eliminating macrophages may reverse the therapeutic potential of combined NETs and Fn14 blockade. To surmise this postulation, the colony stimulating factor 1 receptor antibody (anti-CSF1R) was injected into CLP mice receiving SIVE and/or ITEM-2 therapy to immunodeplete macrophages (Figure 2A, [+]). Anti-CSF1R almost entirely abolished infiltration of macrophages in renal tissues (*P* < 0.01 and *P* < 0.001, Figure S3B & C), leading to a significant impediment in mortality rescue (*P* = 0.0174, Figure 2B) and tubular protection (*P* < 0.001, Figure 2C & D) upon SIVE and ITEM-2 combination therapy. In mice receiving SIVE plus ITEM-2 therapy, anti-CSF1R injection restored production of HMGB1 relative to IL-1β (*P* < 0.001, Figure 2I). We also utilized clodronate liposome (CL) to eliminate circulating macrophages (*P* < 0.001, Figure S5A & B) and found that it shortened median survival time of CLP mice receiving SIVE plus ITEM-2 therapy (*P* = 0.048, Figure S5C) and largely impaired the ability of combination therapy to attenuate tubular damage (*P* < 0.001, Figure S5D & E). On the other hand, elimination of circulating macrophages by CL did not affected Scr (*P* = 0.1047, Figure S5F) and BUN (*P* = 0.0689, Figure S5G) clearance but partially restored HMGB1 (*P* < 0.05, Figure S5H) and IL-1β (*P* < 0.05, Figure S5I) production. Therefore, macrophages are instrumental for the synergistic effects of combined NETs and Fn14 blockade against septic AKI.

### Combined blockade of NETs and Fn14 perpetuates infiltration and survival of GAS6^+^ macrophages

**Figure 3:**
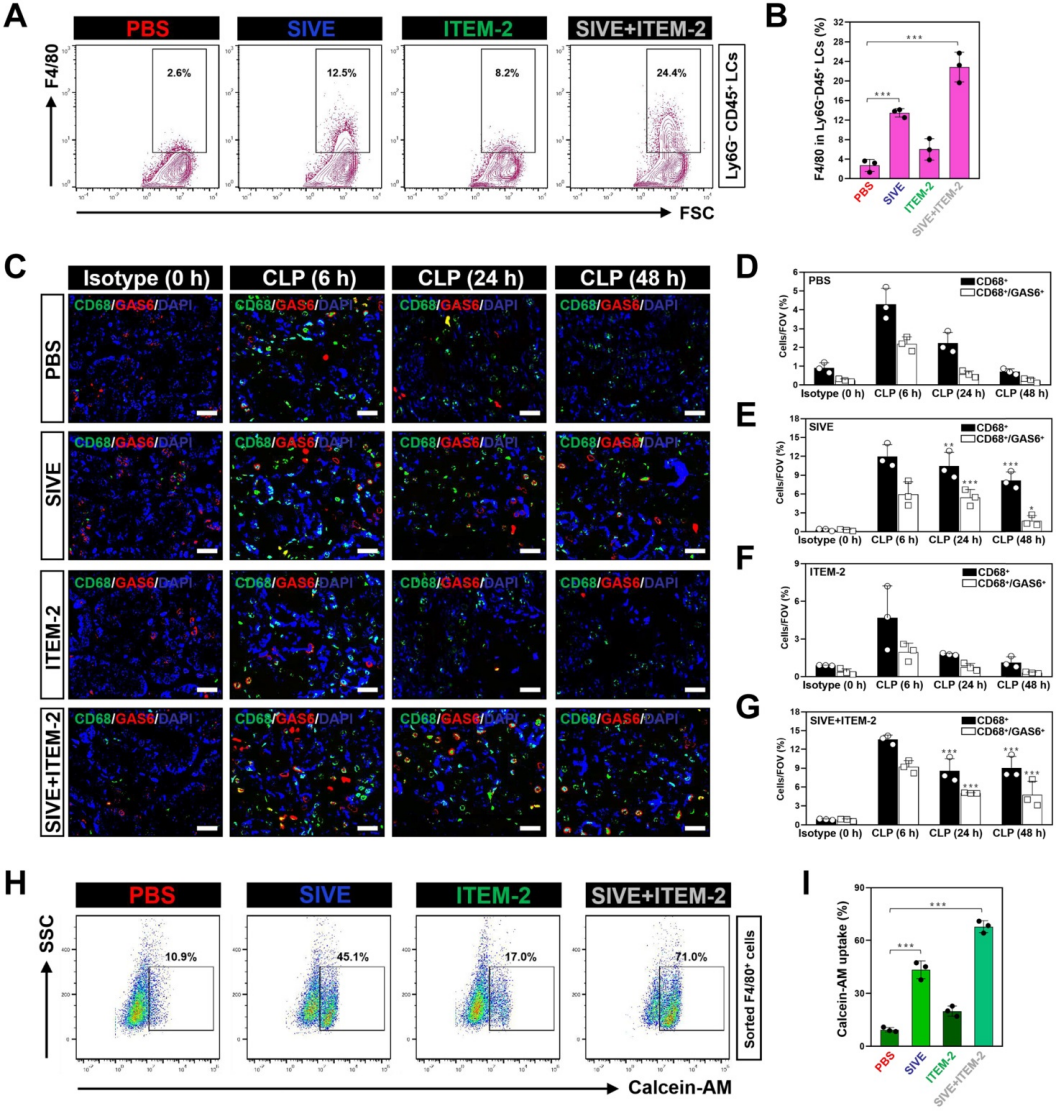
** Combined blockade of NETs and Fn14 perpetuates infiltration and survival of GAS6^+^ macrophages. (A and B)** FACS analyses (A) and histogram (B) evaluating percentage of F4/80^+^ macrophages in Ly6G^-^CD45^+^ live cells (LCs) from kidney tissues of CLP mice with intraperitoneal administration of SIVE, ITEM-2 or both. Data are expressed as mean ± s.d. ***P < 0.001, one-way ANOVA, post hoc comparisons, Tukey's test. **(C)** Representative imags of CD68^+^/GAS6^+^ staining in renal sections from CLP mice with intraperitoneal administration of SIVE, ITEM-2 or both. Scale bar: 50 µm. **(D-G)** Quantification of CD68^+^ and CD68^+^/GAS6^+^ staining intensity in renal sections from CLP mice with intraperitoneal administration of PBS (D), SIVE (E), ITEM-2 (F) or both (G). Data are expressed as mean ± s.d. **P < 0.01 and ***P < 0.001 versus PBS, one-way ANOVA, post hoc comparisons, Tukey's test. **(H and I)** FACS analyses (H) and histogram (I) detecting calcein-AM uptake of renal F4/80^+^ macrophages sorted from CLP mice with intraperitoneal administration of SIVE, ITEM-2 or both. Data are expressed as mean ± s.d. ***P < 0.001, one-way ANOVA, post hoc comparisons, Tukey's test.

The impairment of the anti-septic AKI phenotypes of combined NETs and Fn14 blockade by macrophage elimination prompted us to decipher the role of combination therapy in macrophages. To address this issue, we monitored the changes of macrophages infiltration in kidney tissues from CLP mice receiving SIVE and/or ITEM-2 therapy. Fluorescence-activated cell sorter analysis (FACS) identified higher renal F4/80^+^ population in the SIVE-treated or the SIVE plus ITEM-2-cotreated CLP mice as compared to those in the PBS-treated CLP mice (*P* < 0.001, Figure 3A & B). CLP mice receiving Cl-Amidine monotherapy or Cl-Amidine plus ITEM-2 combination therapy also had more renal F4/80^+^ population in compared with those from counterparts receiving PBS therapy as well (*P* < 0.01, Figure S6A & B). IF analyses further confirmed the FACS observations and showed that greater percentage of CD68^+^ macrophages were present in renal sections of mice at 6-hour time point after CLP when compared with isotype controls (0 h), while they markedly decreased at 24 h and were hardly detectable at 48 h. Of note, SIVE therapy restored infiltration of CD68^+^ macrophages, as did SIVE plus ITEM-2 combination therapy (*P* < 0.01 and *P* < 0.001, Figure 3C-E and G). Restoration of CD68^+^ macrophages infiltration by SIVE was accompanied by an increase in staining intensity of growth arrest-specific protein 6 (GAS6), the ligand bridging macrophages to apoptotic cells for efferocytosis 28, 29, which was also occurred upon the combined SIVE and ITEM-2 therapy (*P* < 0.05 and *P* < 0.001, Figure 3C-G), suggesting that NETs blockade by itself or in combination with Fn14 mAb increases macrophages infiltration and sustains their efferocytosis during septic AKI. To understand the underlying mechanism of how NETs influences macrophages infiltration, we sorted the renal F4/80^+^ macrophages of CLP mice receiving single or combined therapy and employed FACS analyses with calcein-AM staining to determine their cell viability. Figure 3H & I depicted that the calcein-AM uptake of F4/80^+^ macrophages from the SIVE-treated or SIVE plus ITEM-2-cotreated CLP mice were much more than those from the PBS-treated CLP mice (*P* < 0.001). These results indicate that combined NETs and Fn14 blockade perpetuates infiltration and survival of macrophages, which produce copious quantities of GAS6 and thereby reverses the immunosuppressive status of septic AKI.

Take into account that T cells and monocytes are critical for immune modulation of sepsis 30, 31, we determined whether NETs blockade in combination with Fn14 mAb induced changes of the two lineages during septic AKI. Combination therapy with both SIVE and ITEM-2 reduced infiltration of CD4^+^/FOXP3^+^ regulatory T cells (Tregs) (*P* < 0.001, Figure S6C & D) but had negligible effects on infiltration of CD3^+^ total or CD8^+^ cytotoxic T cells (Figure S6E-H). SIVE increased infiltration of CD115^+^ monocytes in combination with or without ITEM-2. There were no significant alterations in Tregs, CD3^+^ total or CD8^+^ cytotoxic T cells infiltration of these groups after elimination of macrophages. In stark contrast, infiltration of CD115^+^ monocytes from the macrophages-eliminated CLP mice were much less than those from the macrophages-intact CLP mice in all groups tested (*P* < 0.01 and *P* < 0.001, Figure S6I & J). Either Cl-Amidine monotherapy or combined Cl-Amidine and ITEM-2 therapy barely influenced infiltration of Tregs and CD115^+^ monocytes (data not shown). These results suggest that combined NETs and Fn14 blockade may partially reprogramme the immunosuppressive renal microenvironment by controlling infiltration of pleiotropic leukocytes such as Tregs and monocytes to underlie the protective mechanism against septic AKI.

To definitively clarify whether restoring activity of macrophages through the combined NETs and Fn14 blockade is sufficient to resolve septic AKI *in vivo*, we harvested F4/80^+^ macrophages from CLP mice receiving SIVE monotherapy or SIVE plus ITEM-2 combination therapy and adoptively transferred them to mice after CLP challenge (Figure S7A). To avoid the interference of endogenous macrophages, mice were pretreated with anti-CSF1R. Adoptive transfer of macrophages from the SIVE-treated or SIVE plus ITEM-2-cotreated mice mitigated tubular damage and synergized with ITEM-2, whereas the parental macrophages from the PBS-treated mice were not efficacious (*P* < 0.05 and* P* < 0.01, Figure S7B & C). These data suggest that macrophages contributes to the renal benefits resulting from NETs blockade in combination with Fn14 mAb therapy during septic AKI.

### Genetic ablation of NETs potentiates the therapeutic efficacy of Fn14 mAb against septic AKI

**Figure 4:**
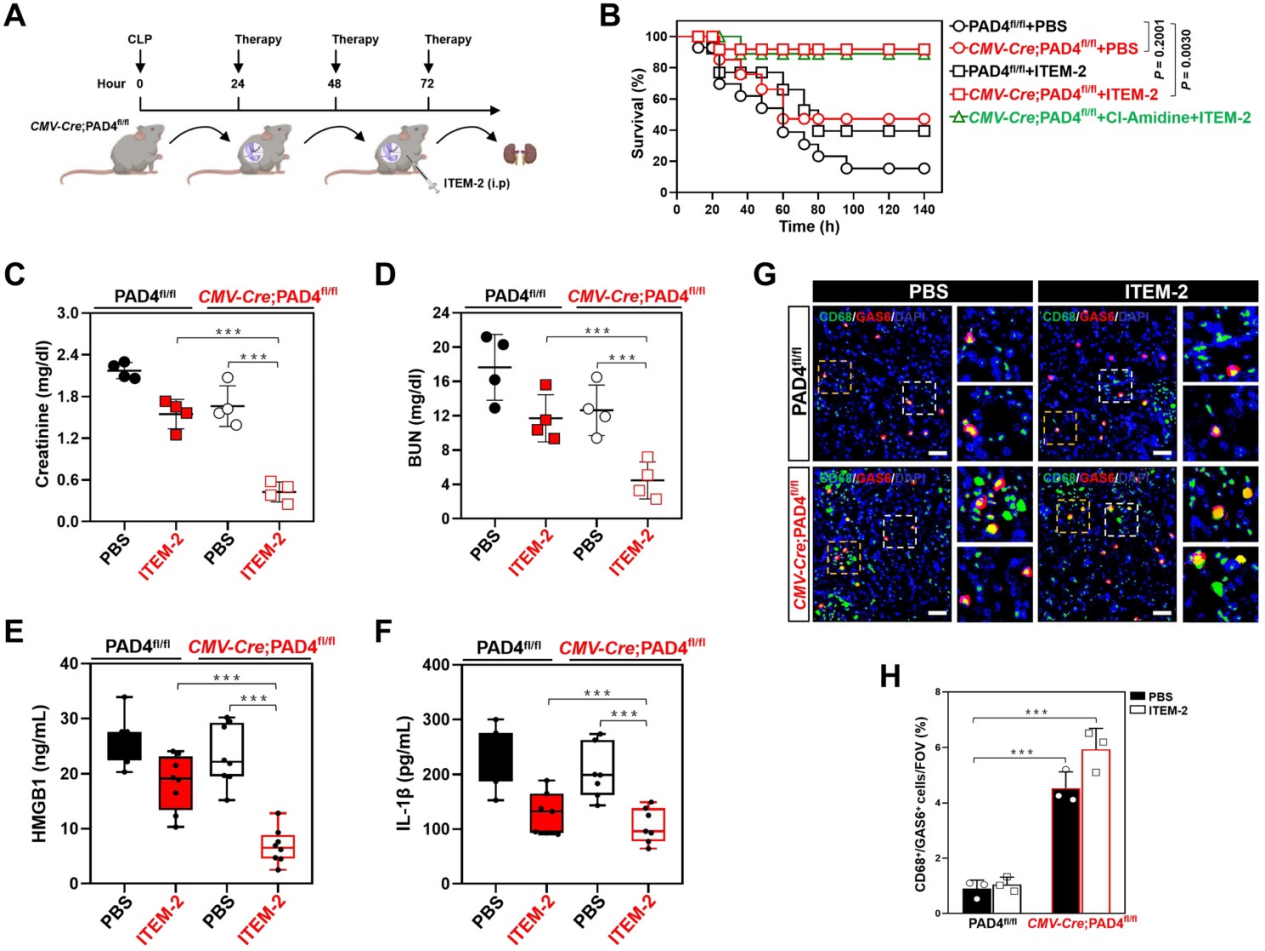
** Genetic ablation of NETs potentiates the therapeutic efficacy of Fn14 mAb against septic AKI. (A)** Experimental scheme of ITEM-2 therapy for *CMV-Cre*; PAD4*^fl/fl^* mice with CLP at the indicated time points. **(B)** Kaplan-Meier curves evaluating survivals of PAD4*^fl/fl^* and *CMV-Cre*; PAD4*^fl/fl^* mice with intraperitoneal administration of PBS or ITEM-2 in the presence or absence of Cl-Amidine treatment at the indicated times after CLP challenge. Log-rank t test was used to caculate the *P* value. **(C and D)** Serum creatinine (Scr, C) and blood urea nitrogen (BUN, D) levels of PAD4*^fl/fl^* and *CMV-Cre*; PAD4*^fl/fl^* mice with intraperitoneal administration of PBS or ITEM-2 upon CLP challenge. Data are expressed as mean ± s.d. **P < 0.01 and ***P < 0.001, one-way ANOVA, post hoc comparisons, Tukey's test. **(E and F)** ELISA assays detecting serum concentrations of HMGB1 (E) and IL-1β (F) in PAD4*^fl/fl^* and *CMV-Cre*; PAD4*^fl/fl^* mice with intraperitoneal administration of PBS or ITEM-2 upon CLP challenge. Data are expressed as mean ± s.d. ***P < 0.001, one-way ANOVA, post hoc comparisons, Tukey's test. **(G and H)** Representative imags (G) and quantification (H) of CD68^+^/GAS6^+^ staining intensity in renal sections of PAD4*^fl/fl^* and *CMV-Cre*; PAD4*^fl/fl^* mice with intraperitoneal administration of PBS or ITEM-2 upon CLP challenge. Data are expressed as mean ± s.d. ***P < 0.001, one-way ANOVA, post hoc comparisons, Tukey's test. Scale bar: 50 µm.

To ask whether the synergistic effects of combined NETs and Fn14 blockade against septic AKI could be recapitulated on a genetic background, the PAD4-deficient *CMV-Cre*; PAD4*^fl/fl^* mice and their PAD4*^fl/fl^* littermates were subjected to CLP, followed by intraperitoneal ITEM-2 injection at 24h after onset of sepsis for a further three days (Figure 4A). Compared with PAD4*^fl/fl^* littermates, the *CMV-Cre*; PAD4*^fl/fl^* mice had little NETs formation irrespective of ITEM-2 injection in the context of CLP (Figure S8A). PAD4 deficiency prolonged survival of CLP mice receiving ITEM-2 injection (*P* = 0.0030) but was unable to do so in CLP mice receiving PBS injection (*P* = 0.2001, Figure 4B). In timescale of ITEM-2 therapy, administering Cl-Amidine to the *CMV-Cre*; PAD4*^fl/fl^* mice did not make them more refractory to mortality caused by CLP, perhaps owing to the fact that little or no druggable target was present in these cases. PAD4 deficiency attenuated septic AKI as reflected by the decrease in serum levels of Scr and BUN, the effects that could be further strengthened by ITEM-2 (*P* < 0.01 and *P* < 0.001, Figure 4C & D). Both HMGB1 and IL-1β production in the *CMV-Cre*; PAD4*^fl/fl^* mice were similar to the PAD4*^fl/fl^* littermates, while they were greatly declined in the presence of ITEM-2 (*P* < 0.001, Figure 4E & F). These results highlight that PAD4 deficiency is required for the host to instill an appropriate innate immunity against septic AKI and that the PAD4 deficiency-mediated NETs interruption may potentiate the anti-septic AKI efficacy of Fn14 mAb.

IF data identified higher population of GAS6^+^ macrophages in renal sections from the *CMV-Cre*; PAD4*^fl/fl^* mice as compared to those from the PAD4*^fl/fl^* littermates upon CLP challenge in the presence or absence of ITEM-2 therapy (*P* < 0.001, Figure 4G & H). Immunodepletion of macrophages using anti-CSF1R deteriorated HMGB1 release of the *CMV-Cre*; PAD4*^fl/fl^* mice receiving ITEM-2 therapy (*P* < 0.01, Figure S8B) during CLP period, along with the increased IL-1β production relative to the control arms (*P* < 0.05, Figure S8C). Therefore, genetic ablation of NETs formation by PAD4 deficiency augments the therapeutic responses of septic AKI to Fn14 mAb through promoting infiltration of GAS6^+^ macrophages.

### NETs blockade transcriptionally upregulates tubular cell-intrinsic Fn14

**Figure 5:**
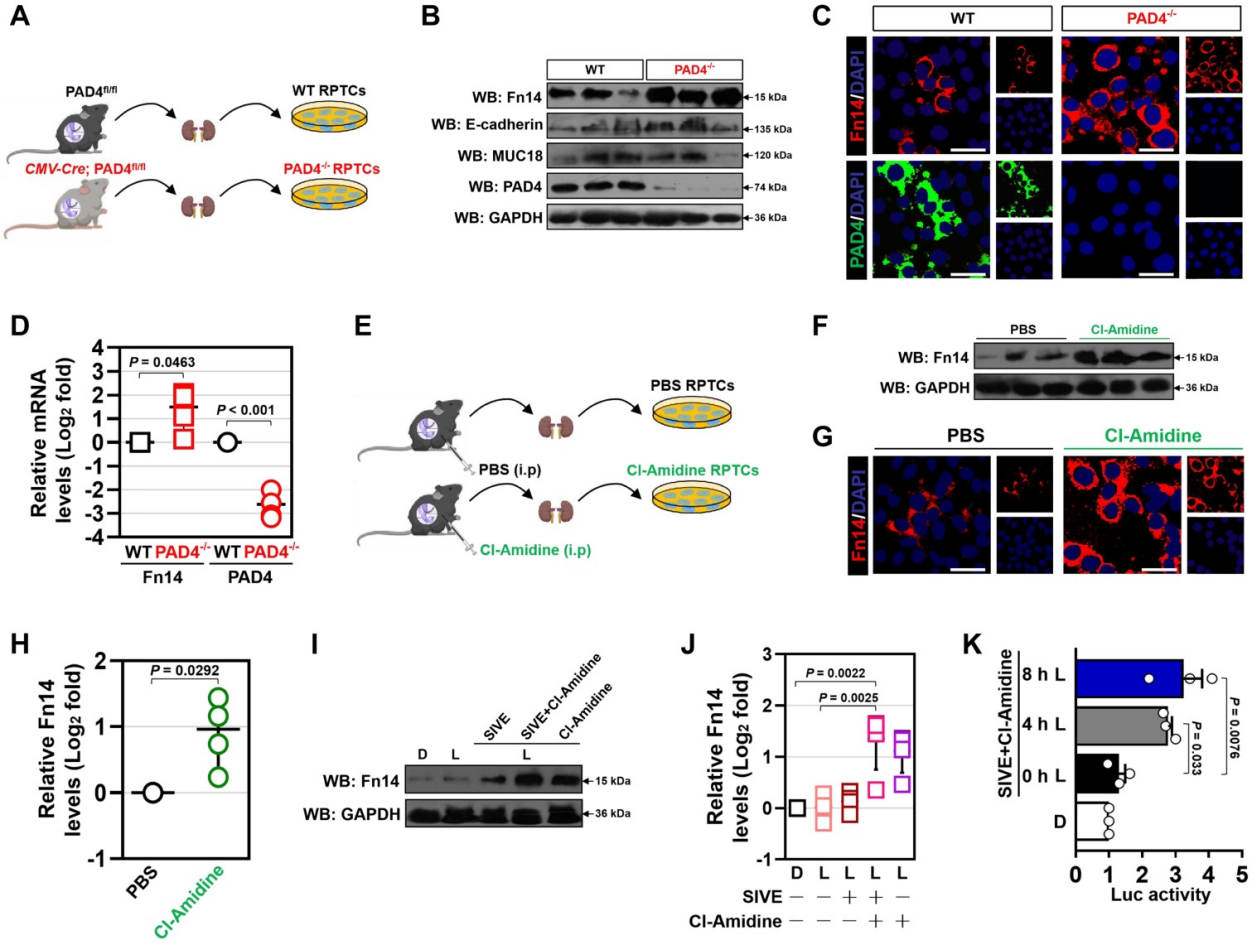
** NETs blockade transcriptionally upregulates tubular cell-intrinsic Fn14. (A)** Experimental scheme showing wild-type (WT) and PAD4^-/-^ renal proximal tubular cells (RPTCs) prepared from renal tissues of PAD4*^fl/fl^* and *CMV*-Cre; PAD4*^fl/fl^* mice upon CLP challenge. **(B)** Western-blotting analyses comparing Fn14, E-cadherin and MUC18 protein abundance in WT and PAD4^-/-^ RPTCs. **(C)** Representative immunfluorescence images detecting membrane accumulation of Fn14 protein in WT and PAD4^-/-^ RPTCs. **(D)** RT-qPCR analyses examining levels of Fn14 mRNA expression in WT and PAD4^-/-^ RPTCs. Experiments were performed four times, each with quantitative RT-PCR in technical duplicate and real-time values were normalized to glyceraldehyde 3-phosphate dehydrogenase (GAPDH). Data are expressed as mean ± s.d. Unpaired, two-tailed Student's *t* test was used to calculate the *P* value. **(E)** Experimental scheme showing RPTCs prepared from renal tissues of mice with PBS or Cl-Amidine administration upon CLP challenge. **(F)** Western-blotting analyses determining Fn14 protein abundance in RPTCs from mice with PBS or Cl-Amidine administration upon CLP challenge. **(G)** Representative immunfluorescence images testing membrane accumulation of Fn14 protein in RPTCs from mice with PBS or Cl-Amidine administration upon CLP challenge. **(H)** RT-qPCR analyses measuring levels of Fn14 mRNA expression in RPTCs from mice with PBS or Cl-Amidine administration upon CLP challenge. Data are expressed as mean ± s.d. Unpaired, two-tailed Student's *t* test was used to calculate the *P* value. **(I)** Western-blotting analyses assessing Fn14 protein levels in the LPS-primed HK-2 cells with SIVE (10 µM), Cl-Amidine (100 µM) or both treatment. D, dimethyl sulfoxide (DMSO). **(J)** RT-qPCR analyses evaluating levels of Fn14 mRNA expression in the LPS-primed HK-2 cells with SIVE, Cl-Amidine or both treatment. Data are expressed as mean ± s.d. One-way ANOVA with Tukey's test post hoc comparisons was used to calculate the *P* value. **(K)** Luciferase assays of the LPS-primed HK-2 cells transfected with full-length Fn14 promoter reporter constructs in the presence of SIVE plus Cl-Amidine cotreatment at the indicate times. Experiments were performed three times and data are expressed as mean ± s.d. One-way ANOVA with Tukey's test post hoc comparisons was used to calculate the *P* value.

Next, we investigated the underlying mechanism of how NETs blockade potentiates the therapeutic efficacy of Fn14 mAb against septic AKI. Since the aformetioned data demonstrated that NETs formation was concurred with Fn14 upregulation during septic AKI, we reasoned NETs may govern tubular cell-intrinsic Fn14 upregulation in this process. To test this possibility, we compared Fn14 expression of renal proximal tubular cells (RPTCs) isolated from PAD4*^fl/fl^* (WT) and *CMV-Cre*; PAD4*^fl/fl^* (PAD4^-/-^) mice with CLP (Figure 5A). To our surprise, the steady-state levels of Fn14 protein were increased, rather than decreased, in the PAD4^-/-^ RPTCs, which had comparable levels of epithelial biomarkers E-cadherin and MUC18 with the WT RPTCs (Figure 5B & C). In parallel with the increased Fn14 protein expression, real-time quantitative reverse transcriptase-polymerase chain reaction (RT-qPCR) with primers specific for messenger RNA (mRNA) of Fn14 delineated that Fn14 mRNA levels were elevated by nearly 1.8-fold in the PAD4^-/-^ RPTCs (*P* = 0.0463, Figure 5D). The protein expression of Fn14 in WT RPTCs increased to the levels of PAD4^-/-^ RPTCs after PAD4 had been knocked out by CRISPR-Cas9 gene editing (Figure S9A). Similar elevation of Fn14 protein and mRNA expression were observed in RPTCs from CLP mice receiving Cl-Amidine therapy (*P* = 0.0292, Figure 5E-H). Cl-Amidine treatment also upregulated Fn14 expression in the LPS-primed human kidney proximal tubular epithelial (HK-2) cells, and such effect remained sustainable even when Cl-Amidine was withdrawn 12 h before analyses (Figure S9B). Despite single SIVE treatment showed a moderate capacity toward Fn14 upregulation in the LPS-primed HK-2 cells, it dramatically increased Fn14 expression in the presence of Cl-Amidine (*P* = 0.0022, Figure 5I & J). However, identical SIVE plus Cl-Amidine cotreatment did not increase expression of DR5 and RANK, other two members of tumor necrosis factor receptor (TNFR) superfamily (*P* = 0.6717 and *P* = 0.9949, Figure S9C & D). To pursue the influence of NETs blockade on transcriptional activity of Fn14, we enrolled a dual-luciferase reporter assay to determine the promoter activity of Fn14 gene in the LPS-primed HK-2 cells expressing pGL3-Fn14-Luc containing the full-length Fn14 promoter. As shown in Figure 5K, SIVE plus Cl-Amidine cotreatment time-dependently enhanced Fn14 promoter activity (*P* = 0.033 and *P* = 0.0076). These results corroborate that blockade of NETs formation transcriptionally upregulates tubular cell-intrinsic Fn14 during septic AKI.

Promoter of *Fn14* gene is hypermetylated by DNA methyltransferase 3a (DNMT3a) 32. We hypothesized that NETs blockade transcriptionally upregulates Fn14 via repressing DNMT3a. Unexpectedly, PAD4 deficiency affected neither protein nor mRNA levels of DNMT3a (Figure S9E & F), and SIVE plus Cl-Amidine cotreatment enhanced Fn14 promoter activity in the DNMT3a-silenced HK-2 cells (*P* = 0.0320, Figure S9G & H). These results suggest that DNMT3a might be dispensable for the transcriptional upregulation of tubular cell-intrinsic Fn14 by NETs blockade.

### HOXA5 stabilization is instrumental for the transcriptional upregulation of Fn14 upon NETs blockade

**Figure 6:**
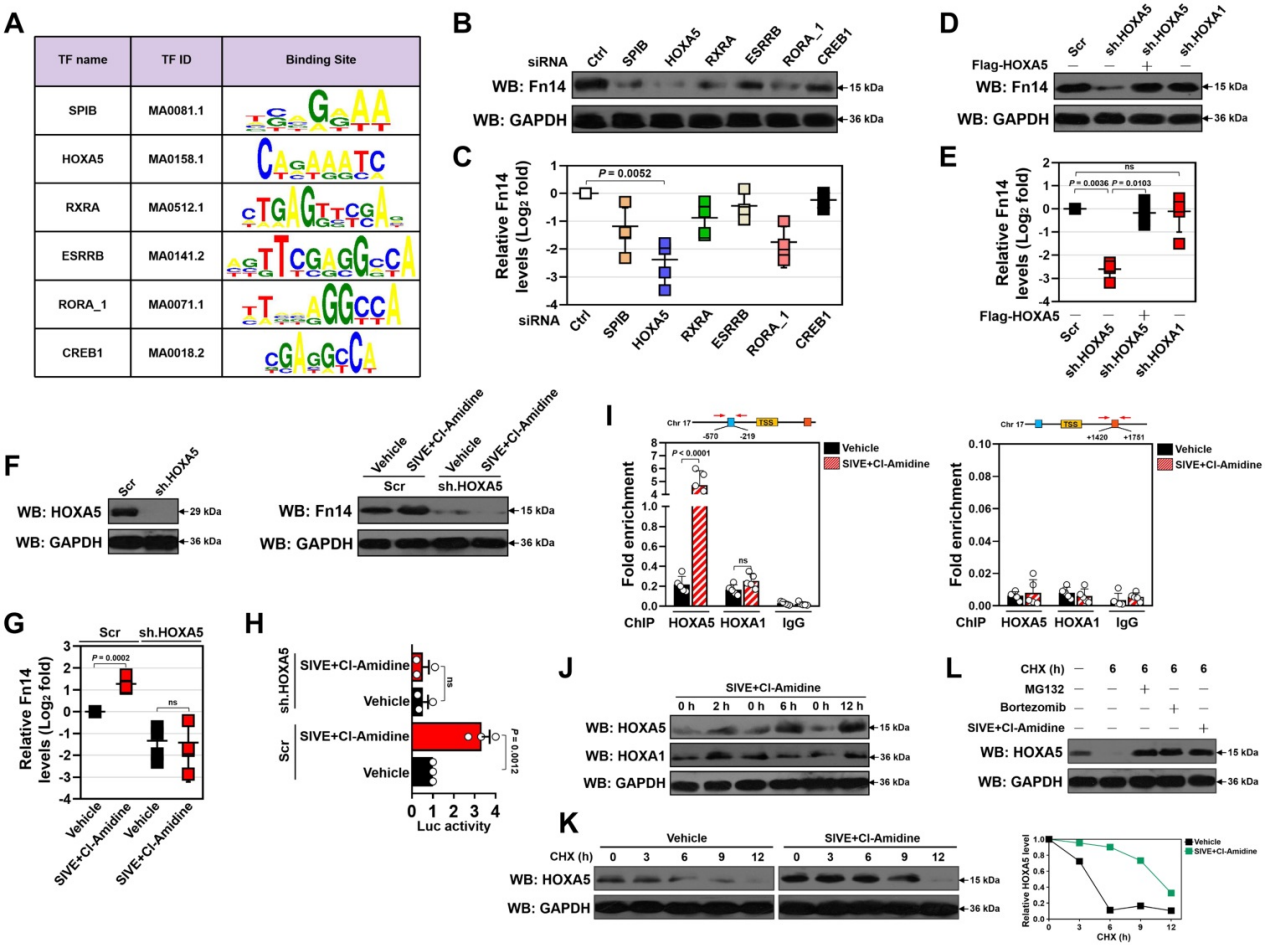
** HOXA5 stabilization is instrumental for the transcriptional upregulation of Fn14 upon NETs blockade. (A)** Known transcription factor (TF)-binding motifs in *Fn14* gene promoter predicted by JASPAR database. The name and ID for each TF are shown. **(B)** Western-blotting analyses comparing Fn14 protein levels in the LPS-primed HK-2 cells with control siRNA (si.Ctrl), SPIB siRNA (si.SPIB), HOXA5 siRNA (si.HOXA5), RXRA siRNA (si.RXRA), ESRRB siRNA (si.ESRBB), RORA_1 siRNA (si. RORA_1) and CREB1 siRNA (si.CREB1) transfection, respectively. **(C)** RT-qPCR analyses evaluating levels of Fn14 mRNA expression in the LPS-primed HK-2 cells with control siRNA (si.Ctrl), SPIB siRNA (si.SPIB), HOXA5 siRNA (si.HOXA5), RXRA siRNA (si.RXRA), ESRRB siRNA (si.ESRBB), RORA_1 siRNA (si. RORA_1) and CREB1 siRNA (si.CREB1) transfection, respectively. Data are expressed as mean ± s.d. One-way ANOVA with Tukey's test post hoc comparisons was used to calculate the *P* value. **(D)** Western-blotting analyses examining Fn14 protein levels in the LPS-primed HK-2 cells with scrambled shRNA (Scr), HOXA5 shRNA (sh.HOXA5) and HOXA1 shRNA (sh.HOXA1) transfection in the presence or absence of the Flag-tagged wild-type HOXA5 re-expression, respectively. **(E)** RT-qPCR analyses assessing levels of Fn14 mRNA expression in the LPS-primed HK-2 cells with scrambled shRNA (Scr), HOXA5 shRNA (sh.HOXA5) and HOXA1 shRNA (sh.HOXA1) transfection in the presence or absence of the Flag-tagged wild-type HOXA5 re-expression, respectively. Data are expressed as mean ± s.d. One-way ANOVA with Tukey's test post hoc comparisons was used to calculate the *P* value. ns, no significant. **(F)** Left panel: Western-blotting analyses determining HOXA5 protein levels in the LPS-primed HK-2 cells with scrambled shRNA (Scr) or HOXA5 shRNA (sh.HOXA5) transfection. Right panel: Western-blotting analyses detecting Fn14 protein levels in the LPS-primed HK-2 cells with scrambled shRNA (Scr) or HOXA5 shRNA (sh.HOXA5) transfection in the presence or absence of SIVE plus Cl-Amidine cotreatment. **(G)** RT-qPCR analyses measuring levels of Fn14 mRNA expression in the LPS-primed HK-2 cells with scrambled shRNA (Scr), or HOXA5 shRNA (sh.HOXA5) transfection in the presence or absence of SIVE plus Cl-Amidine cotreatment. Data are expressed as mean ± s.d. One-way ANOVA with Tukey's test post hoc comparisons was used to calculate the *P* value. ns, no significant. **(H)** Luciferase assays of the LPS-primed HK-2 cells with scrambled shRNA (Scr) or HOXA5 shRNA (sh.HOXA5) transfection in the presence or absence of SIVE plus Cl-Amidine cotreatment. Data are expressed as mean ± s.d. One-way ANOVA with Tukey's test post hoc comparisons was used to calculate the *P* value. **(I)** ChIP analysis for HOXA5, HOXA1 and IgG binding to Fn14 gene promoter in the LPS-primed HK-2 cells with SIVE plus Cl-Amidine cotreatment. Enrichment of promoter region was normalized by input and data are expressed as mean ± s.d. of at least four experiments. Unpaired, two-tailed Student's *t* test was used to calculate the *P* value. ns, no significant. **(J)** Western-blotting analyses testing HOXA5 and HOXA1 protein levels in the LPS-primed HK-2 cells with SIVE plus Cl-Amidine cotreatment at the indicated time points. **(K)** CHX pulse-chase experiments measuring the turnover of HOXA5 protein in the LPS-primed HK-2 cells with SIVE plus Cl-Amidine cotreatment in the presence or absence of 20 µg/mL CHX treatment for the indicated times. (L) Western-blotting analyses comparing HOXA5 protein levels in the LPS-primed HK-2 cells treated with CHX in the presence or absence of MG132 (10 µM) treatment, Bortezomib (1 µM) treatment or SIVE plus Cl-Amidine cotreatment.

In order to dissect the candidate transcription factor (TF) responsible for Fn14 upregulation, we analyzed nucleotide sequence of Fn14 gene promoter region using JASPAR database 33 and identified six putative TFs that may bind directly to and modulate transcriptional activity of Fn14 (Figure 6A). Among them, homeobox protein Hox-A5 (HOXA5) appeared to be the top hit since the most prominent suppression in protein and mRNA expression of Fn14 were observed after silencing HOXA5 with small interfering RNA (siRNA) (*P* = 0.0052, Figure 6B-C and S10A-F). To further determine whether HOXA5 is involved in the NETs blockade-inducible transcriptional upregulation of Fn14, we utilized short hairpin RNA (shRNA) duplexes to deplete HOXA5 in the LPS-primed HK-2 cells before and after SIVE plus Cl-Amidine cotreatment. Depleting HOXA5 in the LPS-primed HK-2 cells led to a significant downregulation in Fn14 protein and mRNA expression that could be ameliorated by reconstituted expression of HOXA5 (*P* = 0.0036 and *P* = 0.0103, Figure 6D & E and S10G). Nevertheless, depletion of HOXA1 had little effect on Fn14 expression (Figure 6D & E and S10H). Upon LPS stimuli, depleting HOXA5 abrogated upregulation of Fn14 protein and mRNA induced by SIVE plus Cl-Amidine cotreatment (Figure 6F & G), which substantially enhanced promoter activity of Fn14 in parental cells but not in cells lacking HOXA5 (Figure 6H). The shRNA-mediated depletion of HOXA5 did not affect cell viability of either the WT or the PAD4^-/-^ RPTCs but normalized high levels of Fn14 in the PAD4^-/-^ RPTCs (*P* = 0.0494, Figure S10I-L). Chromatin immunoprecipitation (ChIP) assays revealed that SIVE plus Cl-Amidine cotreatment increased the recruitment of HOXA5 but not HOXA1 at Fn14 promoter in the LPS-primed HK-2 cells (*P* < 0.0001, Figure 6I) and confirmed the increased HOXA5 occupancy on Fn14 gene promoter in the PAD4^-/-^ RPTCs (*P* = 0.0002, Figure S10M).

The increased recruitment of HOXA5 at Fn14 promoter upon SIVE plus Cl-Amidine cotreatment raises the possibility that NETs formation may function on HOXA5 to regulate Fn14 transcription. Indeed, although no changes of HOXA5 mRNA were observed, SIVE plus Cl-Amidine increased accumulation of HOXA5 protein in the LPS-primed HK-2 cells following 2 h of cotreatment (Figure 6J). However, HOXA1 protein accumulation was heterogeneously influenced by SIVE plus Cl-Amidine cotreatment. HOXA5 protein levels in the PAD4^-/-^ RPTCs were higher than those in WT RPTCs, and exogenous knockout of PAD4 increased accumulation of HOXA5, instead of HOXA1 protein in WT RPTCs (Figure S10N). In support of this, cycloheximide (CHX) pulse-chase experiments showed that CHX gradually accelerated HOXA5 protein turnover in a time-dependent manner, which could be counteracted by SIVE plus Cl-Amidine cotreatment (Figure 6K). HOXA5 also degraded at a much slower rate in the PAD4^-/-^ RPTCs when protein synthesis was inhibited by CHX (Figure S10O). Proteasome inhibitor MG132 or bortezomib blocked the CHX-induced HOXA5 turnover to a similar extent as SIVE plus Cl-Amidine cotreatment did (Figure 6L). These data indicate that proteasomes play important roles in the NETs blockade-inducible HOXA5 stabilization.

### Combination of NETs and Fn14 blockade prevents endotoxemic AKI

**Figure 7:**
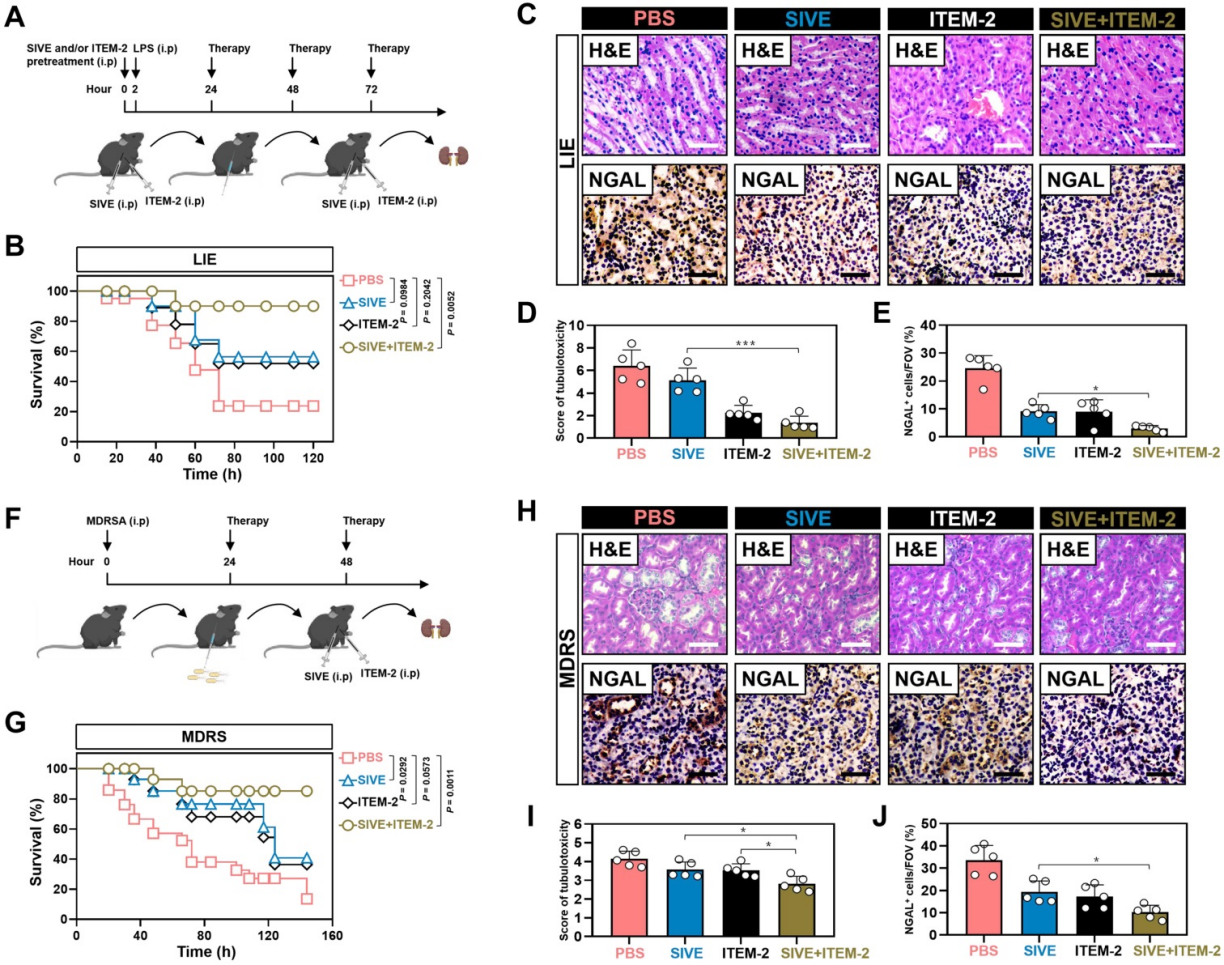
** Combination of NETs and Fn14 blockade prevents AKI in endotoxemic and multidrug-resistant sepsis. (A)** Experimental scheme of SIVE or ITEM-2 monotherapy and SIVE plus ITEM-2 combination therapy for mice receiving intraperitoneal LPS (10 mg/kg) injection at the indicated time points. **(B)** Kaplan-Meier curves analyzing survivals of LIE mice with intraperitoneal administration of SIVE, ITEM-2 or both at the indicated times. Log-rank t test was used to caculate the *P* value. LIE, LPS-induced endotoxemia. **(C-E)** Representative images (C) and quantification for H&E (D) and NGAL (E) staining in renal sections from LIE mice with intraperitoneal administration of SIVE, ITEM-2 or both. Data are expressed as mean ± s.d. *P < 0.05 and ***P < 0.001, one-way ANOVA, post hoc comparisons, Tukey's test. Scale bar: 50 µm. **(F)** Experimental scheme of SIVE or ITEM-2 monotherapy and SIVE plus ITEM-2 combination therapy for mice receiving intraperitoneal MDRSA infection at the indicated time points. **(G)** Kaplan-Meier curves analyzing survivals of MDRS mice with intraperitoneal administration of SIVE, ITEM-2 or both at the indicated times. Log-rank t test was used to caculate the *P* value. MDRS, multidrug-resistant sepsis. **(H-J)** Representative images (H) and quantification for H&E (I) and NGAL (J) staining in renal sections from MDRS mice with intraperitoneal administration of SIVE, ITEM-2 or both. Data are expressed as mean ± s.d. *P < 0.05, one-way ANOVA, post hoc comparisons, Tukey's test. Scale bar: 50 µm.

We next sought to evaluate the therapeutic potential of combined NETs and Fn14 blockade against septic AKI in murine models of LIE. For this purpose, two hours prior to intraperitoneal injection of LPS, mice were administered with SIVE, ITEM-2 and both, respectively, and then 24+, 48+ and 72+ hours after administration, mice were received the same therapies (Figure 7A). In this experiment, LIE mice with either SIVE or ITEM-2 administration did not show statistically significant survival improvement in comparison to the control counterparts with PBS administration (*P* = 0.0984 and *P* = 0.2042), but LIE mice with SIVE and ITEM-2 coadministration did (*P* = 0.0052, Figure 7B). Importantly, LIE mice with the combined SIVE and ITEM-2 therapy had the lowest levels of tubulotoxicity (*P* < 0.01 and *P* < 0.001, Figure 7C-D), and the percentage of cells positive for NGAL staining in their renal sections were greatly dropped (*P* < 0.05, Figure 7C-E). Combined therapy more effectively restored the ability of LIE mice to clear BUN (*P* < 0.05, Figure S11A) and diminish IL-1β (*P* < 0.01 and *P* < 0.05, Figure S11B) production than monotherapy. Single-agent Cl-Amidine showed little capacity in Scr clearance (*P* = 0.5597), whereas Cl-Amidine plus ITEM-2 therapy made LIE mice resistant to tubular damage (*P* < 0.001 and *P* = 0.0054, Figure S12A-C).

### Targeting NETs formation in combination with Fn14 mAb attenuates AKI of multidrug-resistant sepsis

To verify the efficacy of NETs blockade combined with Fn14 mAb further, mice were treated with SIVE, ITEM-2 or both at 24+ and 48+ hours after being intraperitoneally infected with multidrug-resistant *Staphylococcus aureus* (MDRSA) (Figure 7F). MDRS mice with SIVE plus ITEM-2 combination therapy had higher OS in contrast to MDRS mice with SIVE or ITEM-2 monotherapy (*P* = 0.0011, Figure 7G). Combined therapy of SIVE plus ITEM-2 displayed the stronger ability to dampen tubulotoxicity than monotherapy (*P* < 0.05, Figure 7H & I). Renal sections from MDRS mice with SIVE plus ITEM-2 combination therapy also displayed the lowest levels of NGAL-positive staining (*P* < 0.05, Figure 7H-J). Even though we only observed a modest increase in Scr clearance from MDRS mice receiving combination therapy (*P* < 0.05, Figure S13A), their BUN clearance were markedly accelerated as compared with those from MDRS mice receiving monotherapy (*P* < 0.01, Figure S13B). Combined therapy of SIVE and ITEM-2 more profoundly impaired HMGB1 (*P* < 0.01, Figure S13C) and IL-1β (*P* < 0.05, Figure S13D) production than single SIVE therapy. In addition, Cl-Amidine in combination with ITEM-2 therapy prolonged OS of MDRS mice (*P* < 0.001, Figure S14A) and protected them against septic AKI, which were more evident than Cl-Amidine therapy alone (*P* < 0.01 and *P* < 0.001, Figure S14B-D).

## Discussion

Septic AKI is conventionally viewed as a stubborn clinical syndrome due to the severe, uncontrollable inflammation, with no satisfactory treatments widely available hitherto. Patients with septic AKI often progress to immunosuppressive status after being suffered from the initial systemic inflammatory response syndrome (SIRS), which is characterized by imbalance of inflammation-immune network, which leads to disruption in host immunity, paralysis to eradicate pathogens and preference to tubular damage 34. NETs are thought to be a double-edged sword for host defense against microbiota. That is, although NETs formation provides a physical fibrous scaffold to entangle bacteria or pathogens, excessive NETs activation deteriorates inflammation and tissue injury 35. Our recent study demonstrated the involvement of Fn14 in the LPS-inducible tubular damage and that deactivating tubular Fn14 by ITEM-2 prevents septic AKI 17. Based on these hints and evidences, we hypothesize that NETs blockade may reprogramme immunological features of septic AKI, thereby potentiating the therapeutic efficacy of ITEM-2.

In the current study, we demonstrate that NETs are enriched in murine AKI of three distinct sepsis procedures, including CLP, LIE and MDRS, which concurs with Fn14 upregulation. Both serum NETs and Fn14 levels are found to be elevated in clinical patient with septic AKI, thus reinforcing our findings in animals. Pharmacological blockade of NETs formation using SIVE or Cl-Amidine dampens septic AKI and potentiates the therapeutic efficacy of ITEM-2. Combined NETs and Fn14 blockade perpetuates survival of efferocytic GAS6^+^ macrophages and sustains their infiltration in kidneys. This is significant, as elimination of macrophages reverses the beneficial effects of combined therapy. We note that NETs formation is disrupted in CLP mice lacking PAD4, which display improvement in survival and impairment in septic AKI upon ITEM-2 therapy. Intriguingly, biochemical and genetic analyses reveal that NETs blockade dismantles the proteasomes-mediated turnover of HOXA5, which directly binds to Fn14 gene promoter, resulting in the transcriptional upregulation of Fn14. Both LIE and MDRS mice with combined NETs and Fn14 blockade show lesser degree of AKI than those with single-agent therapy. Our future work will aim at exploring the mechanistic linkage of NETs formation with tubular cell-intrinsic Fn14 signalling during septic AKI and ascertaining whether additional sepsis-related organ dysfunction would also be suitable for such combination therapy.

Aberrant NETs formation has been observed in pathogenesis of acute and chronic inflammatory diseases, including sepsis 36, ulcerative colitis 37, rheumatoid arthritis 38 and vasculitis 39. Therefore, proper control of NETs formation is pivotal for preventing development of these diseases. In fact, persistent inflammatory responses are associated with inappropriate recruitment and activation of neutrophils, which constitutes an important factor for tissue injury 40. Our data suggest that NETs are enriched during septic AKI and such enrichment is synchronized with upregulation of Fn14, which functions as a sensor of pathogen-associated molecular patterns (PAMPs) for evoking transduction of diverse pro-inflammatory routes.

Septic AKI is strikingly immunosuppressive 41, and more data concerning relevance of the immunosuppressive renal microenvironment to its manipulator, such as NETs, are needed. Discovering novel way of immune checkpoint blockade would be helpful to enhance the efficacy of conventional therapy, especially in septic AKI where little approach is available. Before blindly moving forward with a combination strategy, it is necessary to implement scheme based on empirical data and assess the significance of blocking each factor in host. NETs as an immunotherapeutic target have attracted much attention for a number of reasons. First, higher plasma levels of NETs in patients with sepsis correlates with worse overall survival 10. Moreover, NETs formation facilitates intravascular coagulation and sepsis 42. Last but not least, NETs compromises immunity by directing activity of innate immune cells 6. These literature provide preclinical rationales for a principal role of NETs formation in restraining host immunity. On the other hand, the limited efficacy of Fn14 mAb against septic AKI observed in our recent study has prompted further investigation into combination therapy. We currently demonstrate that combined therapy of either the NE inhibitor SIVE or the PAD4 inhibitor Cl-Amidine with Fn14 mAb synergistically provides protection against AKI in distinct murine models of sepsis. We propose a therapeutic strategy that interruption of NETs formation reprograms host defense and simultaneously makes tubular cells more additive to the intrinsic Fn14 signalling, thereby potentiating their responsiveness to Fn14 mAb. The next step for logistical translation will be to understand whether this combination therapy works in the context of or prior to first-line antibiotics, the most prevalent anti-septic agents for clinical patients.

Macrophages belong to potent pathogen scavengers responsible for a wide range of infections 43. They constitute an unique population of resident phagocytes that limit inflammation dissemination. Lack of macrophages infiltration is considered to be a main factor of insufficient antimicrobial defense in the inflammatory injury mediated by neutrophils 44. Restoring function and activity of macrophages to their original levels enables more efficient defense than targeting a single cascade as the latter might concomitantly activate other immunosuppressive pathways. In this study, the increased infiltration and survival of efferocytic GAS6^+^ macrophages are observed in renal sections of CLP mice after NETs blockade in combination with or without ITEM-2. Adoptive transfer of macrophages harvested from CLP mice receiving SIVE monotherapy or SIVE plus ITEM-2 combination therapy attenuates tubular damage and synergizes with ITEM-2 in recipients with CLP. Our data complement other event such as clearance of the apoptotic neutrophils, which is triggered by developmental endothelial locus-1 (DEL-1), to restrict inflammation deterioration 45.

To our knowledge, we believe that our study for the first time to identify the impact of NETs interruption on tubular Fn14 transcription during septic AKI. We find that genetic PAD4 ablation upregulates rather than downregulates protein and mRNA expression of Fn14. Pharmacological NETs interruption phenocopies PAD4 deficiency to increase Fn14 abundance. Extending these findings, we observe the augmented promoter activity of *Fn14* gene in HK-2 cells upon SIVE plus Cl-Amidine cotreatment and in RPTCs derived from *CMV-Cre*; PAD4*^fl/fl^* mice. These results lead us to conclude that NETs interruption enhances addiction of tubular cells to the inflammation-dependent, intrinsic Fn14 signalling and thus increases their sensitivities to Fn14 mAb therapy. Despite the DNMT3a-mediated promoter methylation is assumed to repress Fn14 transcription, and our finding that silencing DNMT3a increases the transcriptional activity of Fn14 is consistent with this notion, we found no changes in Fn14 promoter activity after silencing DNMT3a upon SIVE plus Cl-Amidine cotreatment. By contrary, identification of HOXA5 as a primary TF of *Fn14* gene increases the repertoire of other transcriptional regulators, such as mothers against decapentaplegic homolog 4 (SMAD4) 46, retinoic acid receptor (RAR) 47 and signal transducer and activator of transcription 5A (STAT5) 48 that are modulated at different pathways. SMAD4 has been linked to the regulation of intercellular tight junction by increasing expression of junctional adhesion molecule (JAM-3) during sepsis 49. Although the exact mechanism remains unclear, taking our findings together with others, these studies support the concept that SMAD4 and HOXA5 may cooperate to determine tubular fate by governing Fn14 transcription during septic AKI. Intriguingly, HOXA5 protein turnover is dismantled when NETs had been interrupted, underscoring the significance of HOXA5, which acts as a downstream effector for NETs blockade, in the therapeutic efficacy of Fn14 mAb.

The first limitation in our work is that besides macrophages, other immune cells might also engage in the anti-septic AKI phenotypes of combined NETs and Fn14 blockade. Despite elimination of macrophages is sufficient to reverse such phenotypes, it remains to be tested precisely whether additional leukocytes participate in this process. The second limitation is that data in the current study are almost obtained from murine models, a definitive conclusion about the synergistic effects of combined therapy in septic AKI would require analyses of cellular experiments *in vitro*. It may be warranted to determine the effect of combination therapy in the LPS-primed tubular cells upon NETs stimuli.

In summary, our findings shed light on the evidence that pharmacological or genetic interruption of NETs formation synergizes with Fn14 mAb to prevent septic AKI by restoring the infiltration and survival of efferocytic GAS6^+^ macrophages and augmenting the tubular cell-intrinsic Fn14 transcription through dismantling the proteasomes-mediated turnover of HOXA5. This regimen provides rationale for further investigation of immune checkpoint therapy in combination with Fn14 mAb in clinical patients and may become a feasible strategy for septic AKI management.

## Supplementary Material

Supplementary figures and table.

## References

[1] Poston JT, Koyner JL (2019). Sepsis associated acute kidney injury. BMJ.

[2] Gotts JE, Matthay MA (2016). Sepsis: pathophysiology and clinical management. BMJ.

[3] Peerapornratana S, Manrique-Caballero CL, Gomez H, Kellum JA (2019). Acute kidney injury from sepsis: current concepts, epidemiology, pathophysiology, prevention and treatment. Kidney Int.

[4] Mocsai A (2013). Diverse novel functions of neutrophils in immunity, inflammation, and beyond. J Exp Med.

[5] Brinkmann V, Reichard U, Goosmann C, Fauler B, Uhlemann Y, Weiss DS (2004). Neutrophil extracellular traps kill bacteria. Science.

[6] Papayannopoulos V (2018). Neutrophil extracellular traps in immunity and disease. Nat Rev Immunol.

[7] Fousert E, Toes R, Desai J (2020). Neutrophil Extracellular Traps (NETs) Take the Central Stage in Driving Autoimmune Responses. Cells.

[8] Delgado-Rizo V, Martinez-Guzman MA, Iniguez-Gutierrez L, Garcia-Orozco A, Alvarado-Navarro A, Fafutis-Morris M (2017). Neutrophil Extracellular Traps and Its Implications in Inflammation: An Overview. Front Immunol.

[9] Gupta S, Kaplan MJ (2016). The role of neutrophils and NETosis in autoimmune and renal diseases. Nat Rev Nephrol.

[10] Colon DF, Wanderley CW, Franchin M, Silva CM, Hiroki CH, Castanheira FVS (2019). Neutrophil extracellular traps (NETs) exacerbate severity of infant sepsis. Crit Care.

[11] Okeke EB, Louttit C, Fry C, Najafabadi AH, Han K, Nemzek J (2020). Inhibition of neutrophil elastase prevents neutrophil extracellular trap formation and rescues mice from endotoxic shock. Biomaterials.

[12] Di Martino L, Osme A, Kossak-Gupta S, Pizarro TT, Cominelli F (2019). TWEAK/Fn14 Is Overexpressed in Crohn's Disease and Mediates Experimental Ileitis by Regulating Critical Innate and Adaptive Immune Pathways. Cell Mol Gastroenterol Hepatol.

[13] Martin-Sanchez D, Fontecha-Barriuso M, Carrasco S, Sanchez-Nino MD, Massenhausen AV, Linkermann A (2018). TWEAK and RIPK1 mediate a second wave of cell death during AKI. Proc Natl Acad Sci U S A.

[14] Sharif MN, Campanholle G, Nagiec EE, Wang J, Syed J, O'Neil SP (2016). Soluble Fn14 Is Detected and Elevated in Mouse and Human Kidney Disease. PLoS One.

[15] Claus M, Herro R, Wolf D, Buscher K, Rudloff S, Huynh-Do U (2018). The TWEAK/Fn14 pathway is required for calcineurin inhibitor toxicity of the kidneys. Am J Transplant.

[16] Wu J, Min X, Wang L, Yang J, Wang P, Liu X (2018). Fn14 Deficiency Ameliorates Anti-dsDNA IgG-Induced Glomerular Damage in SCID Mice. J Immunol Res.

[17] Mo SJ, Zhang W, Liu JQ, Chen MH, Xu L, Hong J (2019). Regulation of Fn14 stability by SCF(Fbxw7alpha) during septic acute kidney injury. Am J Physiol Renal Physiol.

[18] Hong J, Hu BC, Xu L, Zheng Y, Shao ZQ, Zhang R (2020). MicroRNA-19a Targets Fibroblast Growth Factor-Inducible Molecule 14 and Prevents Tubular Damage in Septic AKI. Anal Cell Pathol (Amst).

[19] Chen ZD, Hu BC, Shao XP, Hong J, Zheng Y, Zhang R (2021). Ascorbate uptake enables tubular mitophagy to prevent septic AKI by PINK1-PARK2 axis. Biochem Biophys Res Commun.

[20] Hu BC, Wu GH, Shao ZQ, Zheng Y, Liu JQ, Zhang R (2020). Redox DAPK1 destabilizes Pellino1 to govern inflammation-coupling tubular damage during septic AKI. Theranostics.

[21] Song YK, Hu BC, Xu L, Liu JQ, Chen X, Zheng Y (2019). Productive transcription of miR-124-3p by RelA and RNA polymerase II directs RIP1 ubiquitination-dependent apoptosis resistance during hypoxia. Exp Cell Res.

[22] Xu L, Jia Y, Yang XH, Han F, Zheng Y, Ni Y (2017). MicroRNA-130b transcriptionally regulated by histone H3 deacetylation renders Akt ubiquitination and apoptosis resistance to 6-OHDA. Biochim Biophys Acta Mol Basis Dis.

[23] Chen ZD, Xu L, Tang KK, Gong FX, Liu JQ, Ni Y (2016). NF-kappaB-dependent transcriptional upregulation of cyclin D1 exerts cytoprotection against hypoxic injury upon EGFR activation. Exp Cell Res.

[24] Wang Y, Zhu J, Liu Z, Shu S, Fu Y, Liu Y (2021). The PINK1/PARK2/optineurin pathway of mitophagy is activated for protection in septic acute kidney injury. Redox Biol.

[25] Wang Y, Wysocka J, Sayegh J, Lee YH, Perlin JR, Leonelli L (2004). Human PAD4 regulates histone arginine methylation levels via demethylimination. Science.

[26] Chen L, Zhao Y, Lai D, Zhang P, Yang Y, Li Y (2018). Neutrophil extracellular traps promote macrophage pyroptosis in sepsis. Cell Death Dis.

[27] Lu F, Lan Z, Xin Z, He C, Guo Z, Xia X (2020). Emerging insights into molecular mechanisms underlying pyroptosis and functions of inflammasomes in diseases. J Cell Physiol.

[28] van der Meer JH, van der Poll T, van 't Veer C (2014). TAM receptors, Gas6, and protein S: roles in inflammation and hemostasis. Blood.

[29] Nepal S, Tiruppathi C, Tsukasaki Y, Farahany J, Mittal M, Rehman J (2019). STAT6 induces expression of Gas6 in macrophages to clear apoptotic neutrophils and resolve inflammation. Proc Natl Acad Sci U S A.

[30] Lu L, Barbi J, Pan F (2017). The regulation of immune tolerance by FOXP3. Nat Rev Immunol.

[31] Murray PJ (2018). Immune regulation by monocytes. Semin Immunol.

[32] Tajrishi MM, Shin J, Hetman M, Kumar A (2014). DNA methyltransferase 3a and mitogen-activated protein kinase signaling regulate the expression of fibroblast growth factor-inducible 14 (Fn14) during denervation-induced skeletal muscle atrophy. J Biol Chem.

[33] Fornes O, Castro-Mondragon JA, Khan A, van der Lee R, Zhang X, Richmond PA (2020). JASPAR 2020: update of the open-access database of transcription factor binding profiles. Nucleic Acids Res.

[34] Gomez H, Kellum JA, Ronco C (2017). Metabolic reprogramming and tolerance during sepsis-induced AKI. Nat Rev Nephrol.

[35] Kaplan MJ, Radic M (2012). Neutrophil extracellular traps: double-edged swords of innate immunity. J Immunol.

[36] Castanheira FVS, Kubes P (2019). Neutrophils and NETs in modulating acute and chronic inflammation. Blood.

[37] Dragoni G, De Hertogh G, Vermeire S (2021). The Role of Citrullination in Inflammatory Bowel Disease: A Neglected Player in Triggering Inflammation and Fibrosis?. Inflamm Bowel Dis.

[38] Khandpur R, Carmona-Rivera C, Vivekanandan-Giri A, Gizinski A, Yalavarthi S, Knight JS (2013). NETs are a source of citrullinated autoantigens and stimulate inflammatory responses in rheumatoid arthritis. Sci Transl Med.

[39] Soderberg D, Segelmark M (2018). Neutrophil extracellular traps in vasculitis, friend or foe?. Curr Opin Rheumatol.

[40] Brown KA, Brain SD, Pearson JD, Edgeworth JD, Lewis SM, Treacher DF (2006). Neutrophils in development of multiple organ failure in sepsis. Lancet.

[41] Venet F, Monneret G (2018). Advances in the understanding and treatment of sepsis-induced immunosuppression. Nat Rev Nephrol.

[42] McDonald B, Davis RP, Kim SJ, Tse M, Esmon CT, Kolaczkowska E (2017). Platelets and neutrophil extracellular traps collaborate to promote intravascular coagulation during sepsis in mice. Blood.

[43] Kotas ME, Medzhitov R (2015). Homeostasis, inflammation, and disease susceptibility. Cell.

[44] Uderhardt S, Martins AJ, Tsang JS, Lammermann T, Germain RN (2019). Resident Macrophages Cloak Tissue Microlesions to Prevent Neutrophil-Driven Inflammatory Damage. Cell.

[45] Kourtzelis I, Li X, Mitroulis I, Grosser D, Kajikawa T, Wang B (2019). DEL-1 promotes macrophage efferocytosis and clearance of inflammation. Nat Immunol.

[46] Chen S, Liu J, Yang M, Lai W, Ye L, Chen J (2015). Fn14, a Downstream Target of the TGF-beta Signaling Pathway, Regulates Fibroblast Activation. PLoS One.

[47] Wang S, Jiang W, Chen X, Zhang C, Li H, Hou W (2012). Alpha-fetoprotein acts as a novel signal molecule and mediates transcription of Fn14 in human hepatocellular carcinoma. J Hepatol.

[48] Roos A, Dhruv HD, Peng S, Inge LJ, Tuncali S, Pineda M (2018). EGFRvIII-Stat5 Signaling Enhances Glioblastoma Cell Migration and Survival. Mol Cancer Res.

[49] Zheng S, Lin Z, Liu Z, Liu Y, Wu W (2018). Lipopolysaccharide Mediates the Destruction of Intercellular Tight Junction among Renal Tubular Epithelial Cells via RhoT1/SMAD-4/JAM-3 Pathway. Int J Med Sci.

