# Supplementary material for: Interruption of neutrophil extracellular traps formation dictates host defense and tubular HOXA5 stability to augment efficacy of anti-Fn14 therapy against septic AKI
Source: Theranostics. 2021 Sep 13;11(19):9431–51. doi: 10.7150/thno.61902 (PMC8490525; doi:10.7150/thno.61902)
Supplement: Supplementary file 1 — Supplementary figures and table. [file thnov11p9431s1.pdf]

Interruption of neutrophil extracellular traps formation dictates host defense and tubular HOXA5 stability to augment efficacy of anti-Fn14 therapy against septic AKI

Yin Ni[1], Bang-Chuan Hu[1], Guo-Hua Wu[2], Zi-Qiang Shao[1], Yang Zheng[1], Run Zhang[1], Jun Jin[1], Jun Hong[1], Xiang-Hong Yang[1], Ren-Hua Sun[1], Jin-Quan Liu[1], Shi-Jing Mo[1†]

[1]*Department of Intensive Care Unit, Zhejiang Provincial People's Hospital, People's Hospital of Hangzhou Medical College, Hangzhou 310014, Zhejiang, P.R.China*

[2]*Zhejiang University School of Medicine, Zhejiang University, Hangzhou 310029, Zhejiang, P.R.China*

Running title: Combined blockade of NETs and Fn14 for septic AKI therapy

[†]**Corresponding author:**

*Shi-Jing Mo, MD, PhD

Department of Intensive Care Unit

Zhejiang Provincial People's Hospital

People's Hospital of Hangzhou Medical College

Hangzhou, Zhejiang 310014

P.R. China

Email : moshijing@hmc.edu.cn

ORCID: https://orcid.org/0000-0002-1303-4766

Supplemental Figure legends, Figures S1-15 and Table S1

Figure S1. Validation of septic AKI elicited by CLP, LIE and MDRS models. (A and B) Representative pictures (A) and quantification (B) of H&E staining in renal sections from CLP mice. Data are expressed as mean ± s.d. ***$P < 0.001$, two-sided Student's *t*-test. Scale bar = 50 μm. (C) Representative transmission electron microscopy (TEM) images examining mitochondrial morphology of kidney tubular epithelial cells in CLP mice. Scale bar = 0.2 μm. (D and E) Serum creatinine (Scr, D) and blood urea nitrogen (BUN, E) levels in mice after CLP challenge. Data are expressed as mean ± s.d. ***$P < 0.001$, two-sided Student's *t*-test. (F) Western blotting analyses testing levels of PAD4 and Fn14 protein expression in kidney tissues from isotype control and CLP mice. WB: western-blotting. (G and H) Representative pictures (G) and quantification (H) of H&E staining in renal sections from LIE mice. Data are expressed as mean ± s.d. ***$P < 0.001$, two-sided Student's *t*-test. Scale bar = 50 μm. LIE: LPS-induced endotoxemia. (I) Representative TEM images testing mitochondrial morphology of kidney tubular epithelial cells in LIE mice. Scale bar = 0.2 μm. (J and K) Serum creatinine (Scr, J) and blood urea nitrogen (BUN, K) levels in mice with intraperitoneal LPS (10 mg/kg) injection. Data are expressed as mean ± s.d. ***$P < 0.001$, two-sided Student's *t*-test. (L and M) Representative pictures (L) and quantification (M) of H&E staining in renal sections from MDRS mice. Data are expressed as mean ± s.d. ***$P < 0.001$, two-sided Student's *t*-test. Scale bar = 50 μm. MDRS: multidrug-resistant sepsis. (N) Representative TEM images detecting mitochondrial morphology of kidney tubular epithelial cells in MDRS mice. Scale bar = 0.2 μm (O and P) Serum creatinine (Scr, O) and blood urea nitrogen (BUN, P) levels in mice with intraperitoneal MDRSA infection. Data are expressed as mean ± s.d. *$P < 0.05$ and ***$P < 0.001$, two-sided Student's *t*-test.

Figure S2. Concurrence of NETs formation and Fn14 upregulation in septic AKI elicited by LIE and MDRS models as well as in patients with septic AKI. (A) Experimental scheme of immunohistofluorescence and immunohistochemistry analyses for NETs formation and Fn14 expression in LIE mice at 48 h. (B and C) Representative pictures (B) and quantification (C) of immunohistofluorescence and

immunohistochemistry analyses comparing NETs formation and Fn14 expression in renal sections from LIE mice at the indicated time points. Data are expressed as mean ± s.d. ***P < 0.001 versus 0 h, one-way ANOVA, post hoc comparisons, Tukey's test. Scale bar = 50 μm. (D) Experimental scheme of immunohistofluorescence and immunohistochemistry analyses for NETs formation and Fn14 expression in MDRS mice at 48 h. (E and F) Representative pictures (E) and quantification (F) of immunohistofluorescence and immunohistochemistry analyses comparing NETs formation and Fn14 expression in renal sections from MDRS mice at the indicated time points. Data are expressed as mean ± s.d. **P < 0.01 and ***P < 0.001 versus 0 h, one-way ANOVA, post hoc comparisons, Tukey's test. Scale bar = 50 μm. (G and H) ELISA assays comparing the serum concentration of NETs (G) and Fn14 (H) in 42 healthy controls (HC) and 93 patients with septic AKI (SA). Data are expressed as mean ± s.d. of three independent experiments. (I) Functional association network of PAD4 with components of Fn14 signalling cascade using STRING database.

Figure S3. Validation of NETs blockade by SIVE and macrophages elimination by anti-CSF1R in CLP mice. (A) ELISA assays measuring the serum concentration of NETs in CLP mice with intraperitoneal administration of SIVE, ITEM-2 or both at the indicated time points. (B and C) Representative pictures (B) and quantification (C) of CD68^{+} staining in renal sections from CLP mice with intraperitoneal administration of SIVE, ITEM-2 or both in the absence [−] or presence [+] of anti-CSF1R pretreatment. Data are expressed as mean ± s.d. **P < 0.01 and ***P < 0.001 versus [−], one-way ANOVA, post hoc comparisons, Tukey's test. Scale bar = 50 μm.

Figure S4. Inhibition of PAD4 synergizes with Fn14 mAb to protect mice against septic AKI. (A) Kaplan-Meier curves analyzing survivals of mice with intraperitoneal administration of Cl-Amidine (50 mg/kg), ITEM-2 (0.5 mg) or both at the indicated times after CLP challenge. Log-rank t test was used to caculate the P value. (B and C) Representative pictures (B) and quantification (C) of H&E staining in kidney tissues from CLP mice with intraperitoneal administration of Cl-Amidine, ITEM-2 or both.

Data are expressed as mean ± s.d. ***$P < 0.001$, one-way ANOVA, post hoc comparisons, Tukey's test. Scale bar = 50 μm. (D and E) Serum creatinine (Scr, D) and blood urea nitrogen (BUN, E) levels in CLP mice with intraperitoneal administration of Cl-Amidine, ITEM-2 or both. Data are expressed as mean ± s.d. *$P < 0.05$, **$P < 0.01$ and ***$P < 0.001$, one-way ANOVA, post hoc comparisons, Tukey's test. (F) ELISA assays assessing serum concentration of HMGB1 in CLP mice with intraperitoneal administration of Cl-Amidine, ITEM-2 or both. Data are expressed as mean ± s.d. *$P < 0.05$, one-way ANOVA, post hoc comparisons, Tukey's test. (G) Kaplan-Meier curves analyzing survivals of Fn14-KO mice with intraperitoneal administration of SIVE (100 mg/kg) or Cl-Amidine (50 mg/kg) at the indicated times after CLP challenge. Log-rank t test was used to caculate the *P* value. (H and I) Representative pictures (H) and quantification (I) of H&E staining in kidney tissues from CLP Fn14-KO mice with intraperitoneal administration of SIVE or Cl-Amidine. Data are expressed as mean ± s.d. *$P < 0.05$ and **$P < 0.01$, one-way ANOVA, post hoc comparisons, Tukey's test. Scale bar = 50 μm.

Figure S5. Elimination of circulating macrophages impairs the synergistic effects of combined NETs and Fn14 blockade to prevent septic AKI. (A and B) Representative pictures (A) and quantification (B) of $CD68^+$ staining in spleen sections from CLP mice with intraperitoneal administration of SIVE plus ITEM-2 in the absence or presence of clodronate liposome (CL) treatment. Data are expressed as mean ± s.d. ***$P < 0.001$, one-way ANOVA, post hoc comparisons, Tukey's test. Scale bar = 100 μm. (C) Kaplan-Meier curves examining survivals of mice with intraperitoneal administration of SIVE plus ITEM-2 in the absence or presence of clodronate liposome (CL) treatment at the indicated times after CLP challenge. Log-rank t test was used to caculate the *P* value. (D and E) Representative pictures (D) and quantification (E) of H&E staining in renal sections from CLP mice with intraperitoneal administration of SIVE plus ITEM-2 in the absence or presence of clodronate liposome (CL) treatment. Data are expressed as mean ± s.d. ***$P < 0.001$, one-way ANOVA, post hoc comparisons, Tukey's test. Scale bar = 50 μm. (F and G)

Serum creatinine (Scr, F) and blood urea nitrogen (BUN, G) levels of CLP mice with intraperitoneal administration of SIVE plus ITEM-2 in the absence or presence of clodronate liposome (CL) treatment. Data are expressed as mean ± s.d. (H and I) ELISA assays detecting release of HMGB1 (H) and IL-1β (I) in CLP mice with intraperitoneal administration of SIVE plus ITEM-2 in the absence or presence of clodronate liposome (CL) treatment. Data are expressed as mean ± s.d. *$P < 0.05$, one-way ANOVA, post hoc comparisons, Tukey's test.

Figure S6. Effects of combined NETs and Fn14 blockade on F4/80$^+$ macrophages, CD4$^+$/FOXP3$^+$ regulatory, CD3$^+$ total or CD8$^+$ cytotoxic T cells and CD115$^+$ monocytes. (A and B) FACS analyses (A) and histogram (B) assessing percentage of F4/80$^+$ macrophages in Ly6G$^-$CD45$^+$ live cells (LCs) from kidney tissues of CLP mice with intraperitoneal administration of Cl-Amidine with or without ITEM-2 administration. Data are expressed as mean ± s.d. **$P < 0.01$, one-way ANOVA, post hoc comparisons, Tukey's test. (C and D) Representative imags (C) and quantification (D) of CD4$^+$/FOXP3$^+$ regulatory T cells in renal sections from CLP mice with intraperitoneal administration of SIVE, ITEM-2 or both. Data are expressed as mean ± s.d. ***$P < 0.001$, one-way ANOVA, post hoc comparisons, Tukey's test. Scale bar = 50 μm. (E and F) Representative imags (E) and quantification (F) of CD3$^+$ T cells in renal sections from CLP mice with intraperitoneal administration of SIVE, ITEM-2 or both. Data are expressed as mean ± s.d. Scale bar = 50 μm. (G and H) Representative imags (G) and quantification (H) of CD8$^+$ T cells in renal sections from CLP mice with intraperitoneal administration of SIVE, ITEM-2 or both. Data are expressed as mean ± s.d. Scale bar = 50 μm. (I and J) Representative imags (I) and quantification (J) of CD115$^+$ monocytes in renal sections from CLP mice with intraperitoneal administration of SIVE, ITEM-2 or both in the presence or absence of clodronate liposome (CL) treatment. Data are expressed as mean ± s.d. **$P < 0.01$ and ***$P < 0.001$, two-sided Student's *t*-test. Scale bar = 50 μm.

Figure S7. NETs interruption endows macrophages with anti-tubulotoxic

properties. (A) Experimental scheme depicting adoptive transfer of renal F4/80$^+$ macrophages from CLP mice receiving PBS, SIVE monotherapy or SIVE plus ITEM-2 combination therapy to CLP mice receiving anti-CSF1R pretreatment. (B and C) Representative pictures (B) and quantification (C) of H&E staining in renal sections from CLP mice with adoptive transfer of renal F4/80$^+$ macrophages from CLP mice receiving PBS, SIVE monotherapy or SIVE plus ITEM-2 combination therapy in the presence or absence of ITEM-2 administration. Data are expressed as mean ± s.d. **P* < 0.05 and ***P* < 0.01, one-way ANOVA, post hoc comparisons, Tukey's test. Scale bar = 50 μm.

Figure S8. Genetic ablation of PAD4 abolishes NETs formation and genetic NETs interruption in combination with Fn14 mAb inhibits HMGB1 and IL-1β production dependent of macrophages. (A) ELISA assays testing the serum concentration of NETs in PAD4$^{fl/fl}$ and *CMV-Cre*; PAD4$^{fl/fl}$ mice with or without intraperitoneal administration of ITEM-2 upon CLP challenge. (B and C) ELISA assays detecting serum concentration of HMGB1 (B) and IL-1β (C) in *CMV-Cre*; PAD4$^{fl/fl}$ mice with intraperitoneal administration of ITEM-2 in the presence or absence of anti-CSF1R treatment upon CLP challenge. Data are expressed as mean ± s.d. **P* < 0.05 and ***P* < 0.01, one-way ANOVA, post hoc comparisons, Tukey's test.

Figure S9. NETs blockade transcriptionally upregulates tubular cell-intrinsic Fn14 in a DNMT3a-dispensable manner. (A) Western-blotting analyses detecting Fn14 protein levels in WT and PAD4$^{-/-}$ RPTCs in the presence or absence of PAD4 sgRNA (sg.PAD4) transfection. (B) Western-blotting analyses determining Fn14 protein levels in the LPS-primed HK-2 cells with Cl-Amidine treatment followed by withdrawal at the indicated times. (C) Western-blotting analyses assessing DR5 and RANK protein levels in the LPS-primed HK-2 cells with SIVE plus Cl-Amidine cotreatment. (D) RT-qPCR analyses comparing levels of DR5 and RANK mRNA expression in the LPS-primed HK-2 cells with SIVE plus Cl-Amidine cotreatment. Data are expressed as mean ± s.d. Unpaired, two-tailed Student's *t* test was used to

calculate the *P* value. (E) Western-blotting analyses detecting DNMT3a protein levels in WT and PAD4$^{-/-}$ RPTCs. (F) RT-qPCR analyses measuring levels of DNMT3a mRNA expression in WT and PAD4$^{-/-}$ RPTCs. Data are expressed as mean ± s.d. Unpaired, two-tailed Student's *t* test was used to calculate the *P* value. (G) Western-blotting analyses evaluating DNMT3a protein levels in the LPS-primed HK-2 cells transfected with control siRNA (si.Ctrl) or DNMT3a siRNA (si.DNMT3a). (H) Luciferase assays of the LPS-primed HK-2 cells expressing full-length Fn14 promoter reporter constructs with control siRNA (si.Ctrl) or DNMT3a siRNA (si.DNMT3a) transfection in the presence or absence of SIVE plus Cl-Amidine cotreatment. Experiments were performed three times and data are expressed as mean ± s.d. One-way ANOVA with Tukey's test post hoc comparisons was used to calculate the *P* value.

Figure S10. PAD4 deficiency transcriptionally upregulates Fn14 via stabilizing HOXA5. (A-F) Western-blotting analyses examining SPIB (A), HOXA5 (B), RXRA (C), ESRRB (D), RORA_1 (E) and CREB1 (F) protein levels in the LPS-primed HK-2 cells with control siRNA (si.Ctrl), SPIB siRNA (si.SPIB), HOXA5 siRNA (si.HOXA5), RXRA siRNA (si.RXRA), ESRRB siRNA (si.ESRBB), RORA_1 siRNA (si. RORA_1) or CREB1 siRNA (si.CREB1) transfection. (G) Western-blotting analyses determining HOXA5 protein abundance in the LPS-primed HK-2 cells transfected with HOXA5 shRNA (sh.HOXA5) in the presence or absence of Flag-tagged wild-type HOXA5 re-expression. (H) Western-blotting analyses detecting HOXA1 protein abundance in the LPS-primed HK-2 cells transfected with HOXA5 shRNA (sh.HOXA1). (I and J) Western-blotting analyses testing HOXA5 protein levels in WT and PAD4$^{-/-}$ RPTCs. (K) Trypan blue assay evaluating survival of WT and PAD4$^{-/-}$ RPTCs in the presence or absence of HOXA5 shRNA (sh.HOXA5) transfection at the indicated time points. Experiments were performed three times and data are expressed as mean ± s.d. (L) Western-blotting analyses comparing Fn14 protein levels in WT and PAD4$^{-/-}$ RPTCs in the presence or absence of HOXA5 shRNA (sh.HOXA5) transfection. (M) ChIP analysis for HOXA5 and IgG binding to

Fn14 gene promoter in WT and PAD4$^{-/-}$ RPTCs. Enrichment of promoter region was normalized by input and data are expressed as mean ± s.d. of at least four experiments. Unpaired, two-tailed Student's *t* test was used to calculate the *P* value. (N) Western-blotting analyses detecting HOXA5 and HOXA1 protein abundance in WT and PAD4$^{-/-}$ RPTCs in the presence or absence of PAD4 sgRNA (sg.PAD4) transfection. (O) CHX pulse-chase experiments determining the turnover of HOXA5 protein in WT and PAD4$^{-/-}$ RPTCs.

Figure S11. Combination of NETs and Fn14 blockade improves renal functions in endotoxemic sepsis. (A) Blood urea nitrogen (BUN) levels of LIE mice with intraperitoneal administration of SIVE, ITEM-2 or both. Data are expressed as mean ± s.d. *$P < 0.05$ and **$P < 0.01$, one-way ANOVA, post hoc comparisons, Tukey's test. (B) ELISA assay detecting release of IL-1β in LIE mice with intraperitoneal administration of SIVE, ITEM-2 or both. Data are expressed as mean ± s.d. *$P < 0.05$ and **$P < 0.01$, one-way ANOVA, post hoc comparisons, Tukey's test.

Figure S12. Combination of NETs and Fn14 blockade improves survivals of mice and protects them against AKI in endotoxemic sepsis. (A and B) Representative images (A) and quantification (B) for H&E staining in renal sections from LIE mice with intraperitoneal administration of Cl-Amidine, ITEM-2 or both. Data are expressed as mean ± s.d. ***$P < 0.001$, one-way ANOVA, post hoc comparisons, Tukey's test. Scale bar = 50 μm. (C) Serum creatinine (Scr) levels of LIE mice with intraperitoneal administration of Cl-Amidine, ITEM-2 or both. Data are expressed as mean ± s.d. One-way ANOVA with Tukey's test post hoc comparisons was used to calculate the *P* value.

Figure S13. Combination of NETs and Fn14 blockade improves renal functions in multidrug-resistant sepsis. (A and B) Serum creatinine (Scr, A) and blood urea nitrogen (BUN, B) levels of MDRS mice with intraperitoneal administration of SIVE, ITEM-2 or both. Data are expressed as mean ± s.d. *$P < 0.05$ and **$P < 0.01$,

one-way ANOVA, post hoc comparisons, Tukey's test. (C and D) ELISA assays detecting release of HMGB1 (C) and IL-1β (D) in MDRS mice with intraperitoneal administration of SIVE, ITEM-2 or both. Data are expressed as mean ± s.d. $^*P < 0.05$ and $^{**}P < 0.01$, one-way ANOVA, post hoc comparisons, Tukey's test.

Figure S14. Combination of NETs and Fn14 blockade improves survivals of mice and protects them against AKI in multidrug-resistant sepsis. (A) Kaplan-Meier curves analyzing survivals of MDRS mice with intraperitoneal administration of Cl-Amidine, ITEM-2 or both at the indicated times. Log-rank t test was used to caculate the *P* value. (B-D) Representative images (B) and quantification for H&E (C) and NGAL (D) staining in renal sections from MDRS mice with intraperitoneal administration of Cl-Amidine, ITEM-2 or both. Data are expressed as mean ± s.d. $^{**}P < 0.01$ and $^{***}P < 0.001$, one-way ANOVA, post hoc comparisons, Tukey's test. Scale bar = 50 μm.

Figure S15. Schematic diagram illustrating the synergistic effects and mechanisms of combined NETs and Fn14 blockade against septic AKI in murine models of LIE, CLP and MDRS.

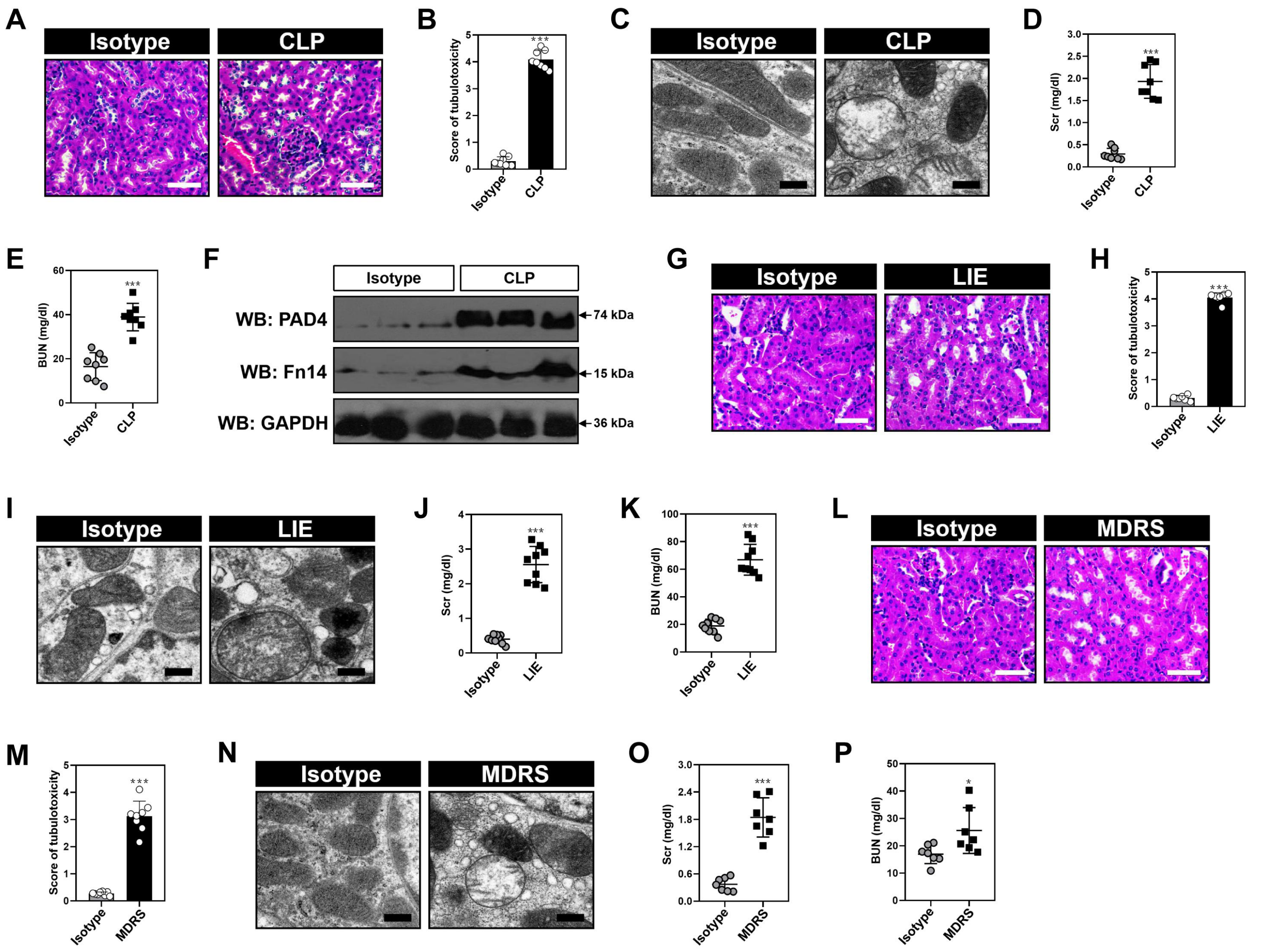

A
Isotype
CLP
B
Score of tubulotoxicity
***
Isotype
CLP
C
Isotype
CLP
D
Scr (mg/dl)
***
Isotype
CLP
E
BUN (mg/dl)
***
Isotype
CLP
F
Isotype
CLP
WB: PAD4
74 kDa
WB: Fn14
15 kDa
WB: GAPDH
36 kDa
G
Isotype
LIE
H
Score of tubulotoxicity
***
Isotype
LIE
I
Isotype
LIE
J
Scr (mg/dl)
***
Isotype
LIE
K
BUN (mg/dl)
***
Isotype
LIE
L
Isotype
MDRS
M
Score of tubulotoxicity
***
Isotype
MDRS
N
Isotype
MDRS
O
Scr (mg/dl)
***
Isotype
MDRS
P
BUN (mg/dl)
*
Isotype
MDRS

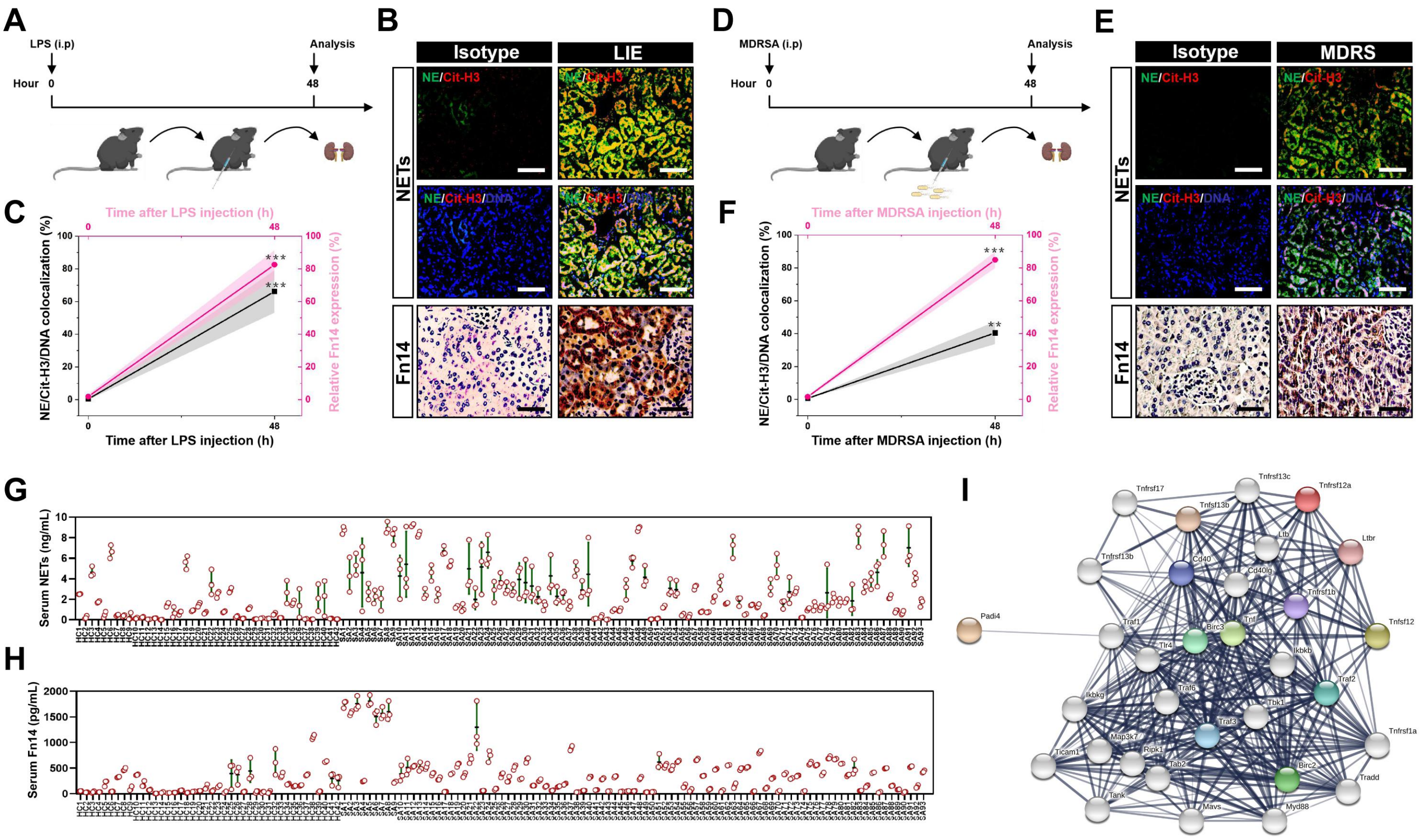
A
LPS (i.p)
Analysis
Hour 0
48
B
Isotype
LIE
NETs
NE/Cit-H3
NE/Cit-H3/DNA
Fn14
C
Time after LPS injection (h)
NE/Cit-H3/DNA colocalization (%)
Relative Fn14 expression (%)
D
MDRSA (i.p)
Analysis
E
Isotype
MDRS
F
Time after MDRSA injection (h)
G
Serum NETs (ng/mL)
H
Serum Fn14 (pg/mL)
I
Tnfrsf17
Tnfrsf13c
Tnfrsf12a
Tnfrsf13b
Ltb
Ltbr
Cd40
Cd40lg
Tnfrsf1b
Padi4
Traf1
Birc3
Tnf
Tnfsf12
Tlr4
Ikbkb
Traf2
Ikbkg
Traf6
Tbk1
Traf3
Map3k7
Tnfrsf1a
Ticam1
Ripk1
Tab2
Birc2
Tradd
Tank
Mavs
Myd88

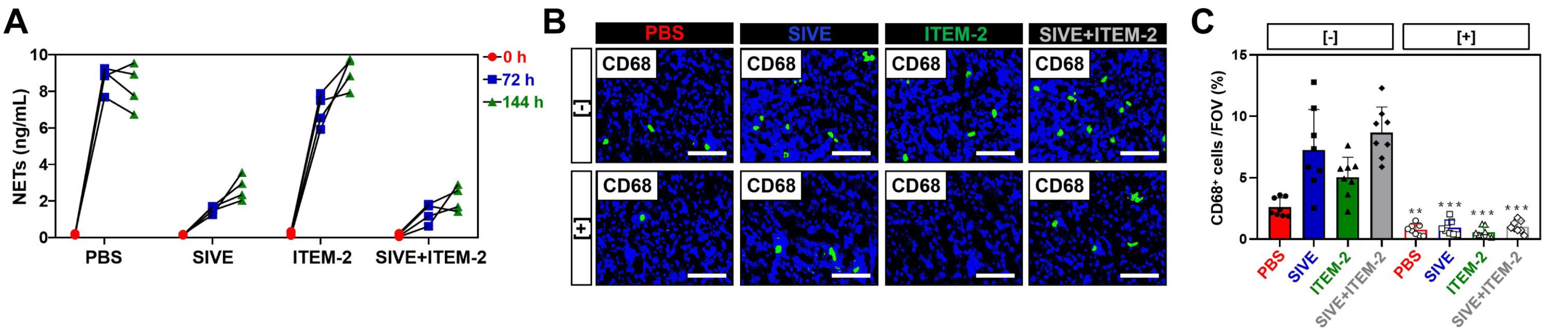

A
NETs (ng/mL)
10
8
6
4
2
0
PBS
SIVE
ITEM-2
SIVE+ITEM-2
0 h
72 h
144 h
B
PBS
SIVE
ITEM-2
SIVE+ITEM-2
[-]
[+]
CD68
C
[-]
[+]
CD68⁺ cells /FOV (%)
15
10
5
0
**
***
***
***
PBS
SIVE
ITEM-2
SIVE+ITEM-2
PBS
SIVE
ITEM-2
SIVE+ITEM-2

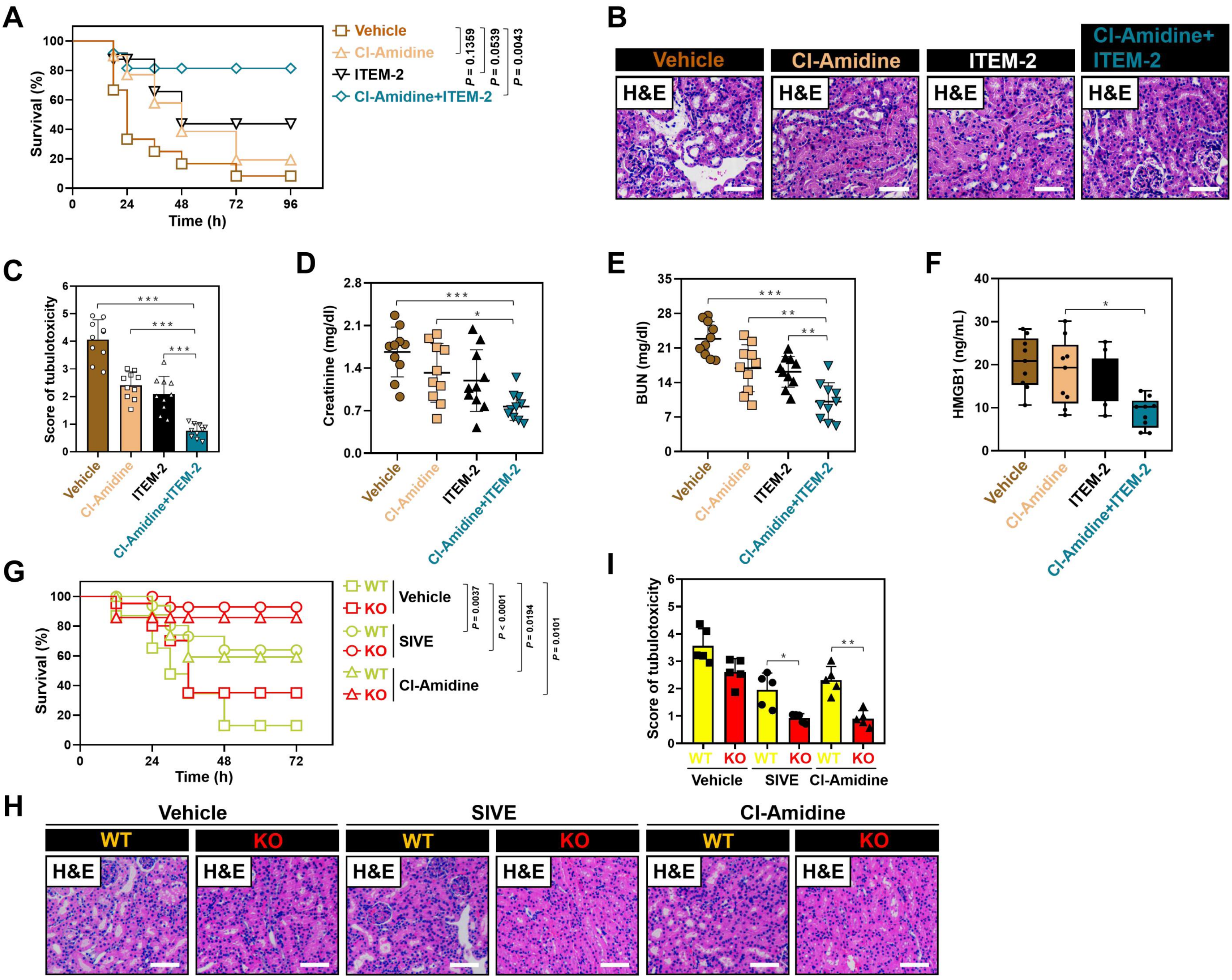

A
Vehicle
Cl-Amidine
ITEM-2
Cl-Amidine+ITEM-2
P = 0.1359
P = 0.0539
P = 0.0043
Survival (%)
Time (h)
B
Vehicle
Cl-Amidine
ITEM-2
Cl-Amidine+ ITEM-2
H&E
C
Score of tubulotoxicity
D
Creatinine (mg/dl)
E
BUN (mg/dl)
F
HMGB1 (ng/mL)
G
WT
KO
Vehicle
SIVE
Cl-Amidine
P = 0.0037
P < 0.0001
P = 0.0194
P = 0.0101
Survival (%)
Time (h)
I
Score of tubulotoxicity
H
Vehicle
SIVE
Cl-Amidine
WT
KO
H&E

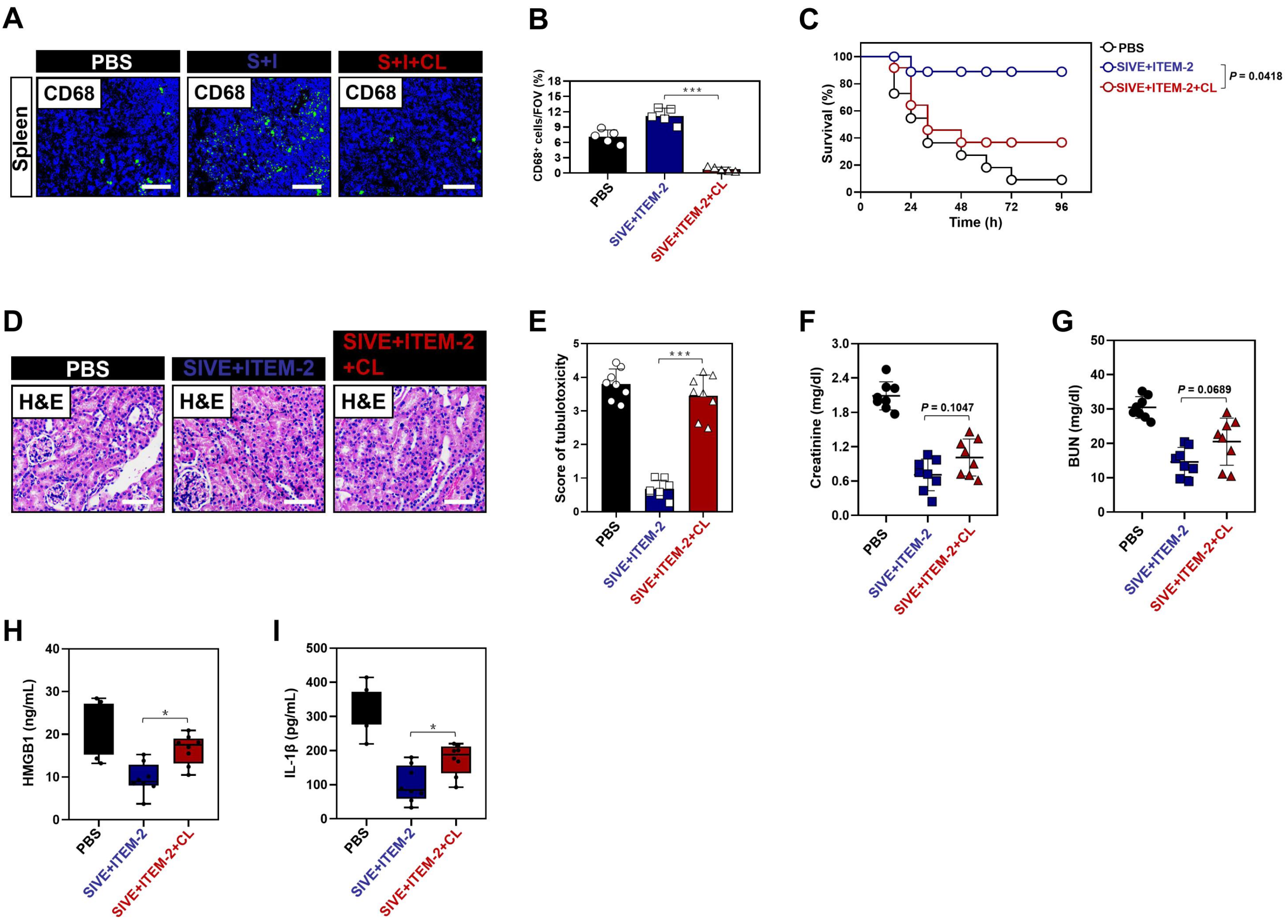
A
PBS
S+I
S+I+CL
Spleen
CD68
B
CD68⁺ cells/FOV (%)
18
15
12
9
6
3
0
***
PBS
SIVE+ITEM-2
SIVE+ITEM-2+CL
C
PBS
SIVE+ITEM-2
SIVE+ITEM-2+CL
P = 0.0418
Survival (%)
100
80
60
40
20
0
0
24
48
72
96
Time (h)
D
PBS
SIVE+ITEM-2
SIVE+ITEM-2 +CL
H&E
E
Score of tubulotoxicity
5
4
3
2
1
0
***
PBS
SIVE+ITEM-2
SIVE+ITEM-2+CL
F
Creatinine (mg/dl)
3.0
2.4
1.8
1.2
0.6
0.0
P = 0.1047
PBS
SIVE+ITEM-2
SIVE+ITEM-2+CL
G
BUN (mg/dl)
50
40
30
20
10
0
P = 0.0689
PBS
SIVE+ITEM-2
SIVE+ITEM-2+CL
H
HMGB1 (ng/mL)
40
30
20
10
0
*
PBS
SIVE+ITEM-2
SIVE+ITEM-2+CL
I
IL-1β (pg/mL)
500
400
300
200
100
0
*
PBS
SIVE+ITEM-2
SIVE+ITEM-2+CL

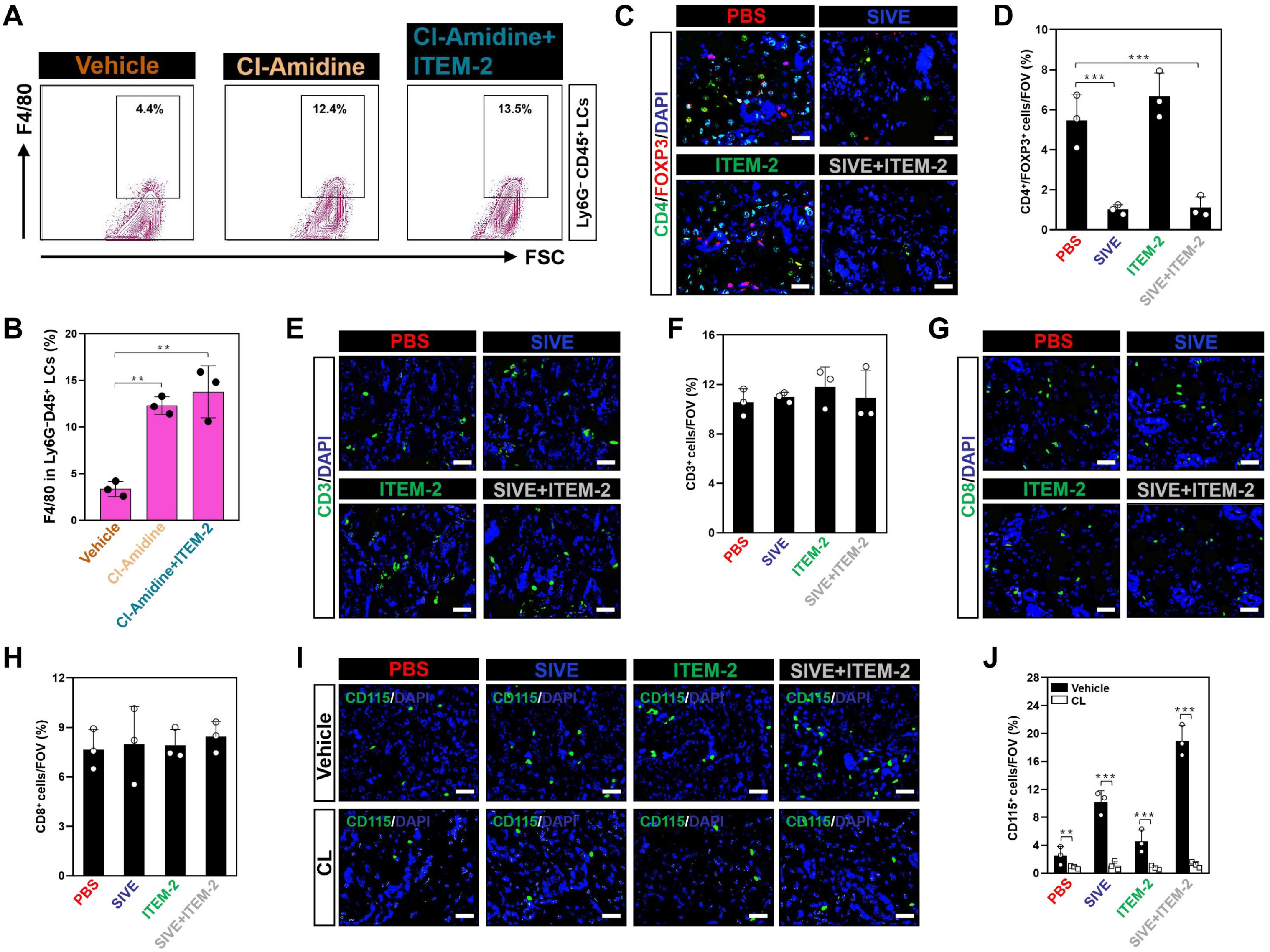
A
Vehicle
Cl-Amidine
Cl-Amidine+ ITEM-2
F4/80
4.4%
12.4%
13.5%
Ly6G⁻ CD45⁺ LCs
FSC
B
F4/80 in Ly6G⁻D45⁺ LCs (%)
Vehicle
Cl-Amidine
Cl-Amidine+ITEM-2
C
PBS
SIVE
ITEM-2
SIVE+ITEM-2
CD4/FOXP3/DAPI
D
CD4⁺/FOXP3⁺ cells/FOV (%)
E
CD3/DAPI
F
CD3⁺ cells/FOV (%)
G
CD8/DAPI
H
CD8⁺ cells/FOV (%)
I
Vehicle
CL
CD115/DAPI
J
Vehicle
CL
CD115⁺ cells/FOV (%)

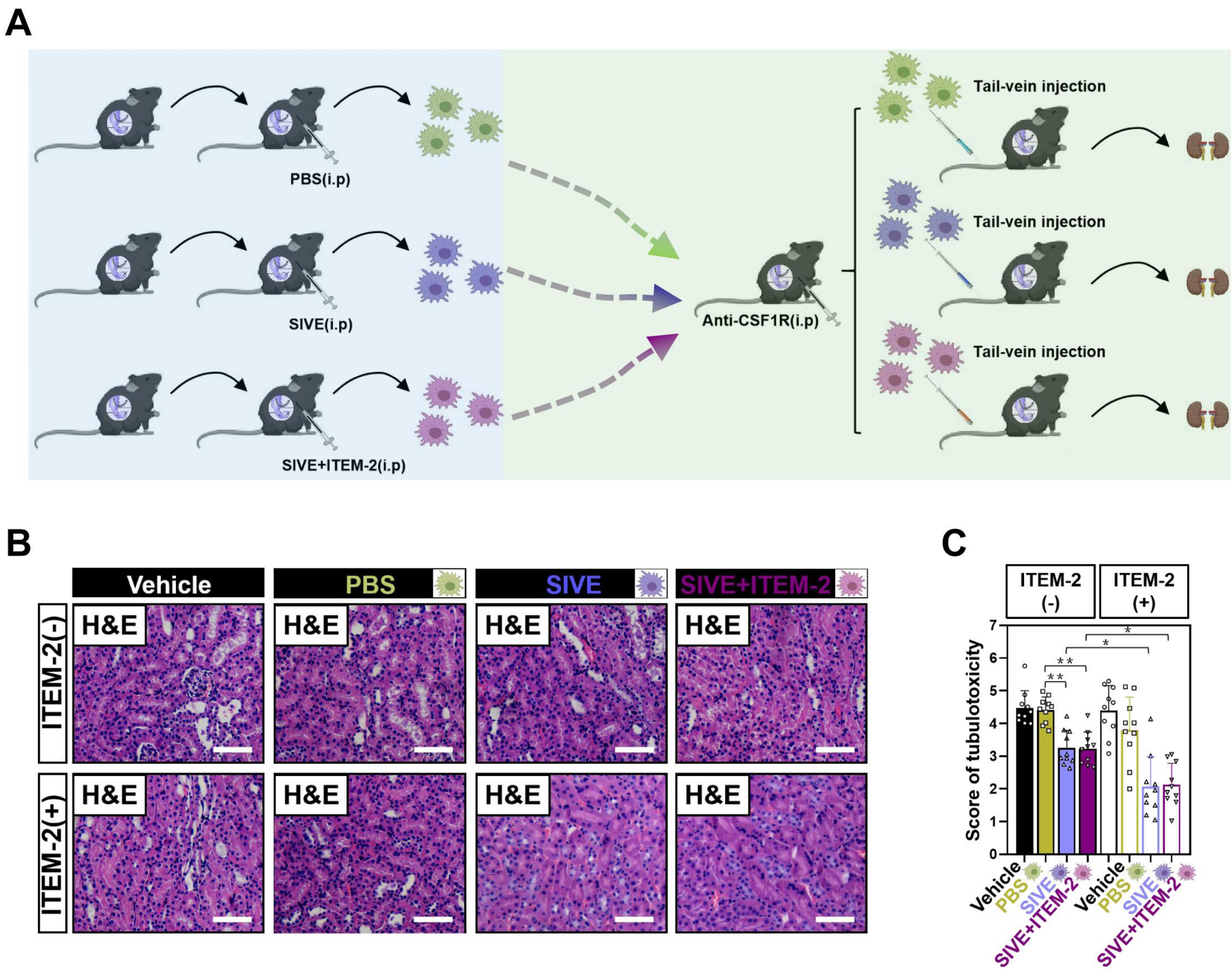
A
PBS(i.p)
SIVE(i.p)
SIVE+ITEM-2(i.p)
Anti-CSF1R(i.p)
Tail-vein injection
Tail-vein injection
Tail-vein injection
B
Vehicle
PBS
SIVE
SIVE+ITEM-2
ITEM-2(-)
ITEM-2(+)
H&E
C
ITEM-2 (-)
ITEM-2 (+)
Score of tubulotoxicity
Vehicle
PBS
SIVE
SIVE+ITEM-2

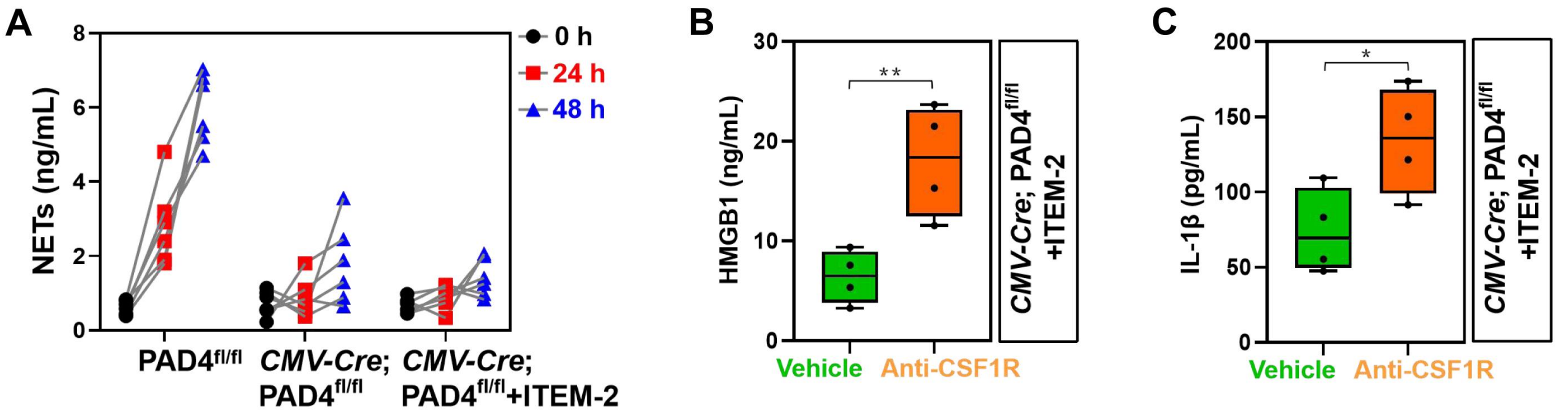

A
NETs (ng/mL)
0 h
24 h
48 h
PAD4fl/fl
CMV-Cre; PAD4fl/fl
CMV-Cre; PAD4fl/fl+ITEM-2
B
HMGB1 (ng/mL)
**
CMV-Cre; PAD4fl/fl +ITEM-2
Vehicle
Anti-CSF1R
C
IL-1β (pg/mL)
*
CMV-Cre; PAD4fl/fl +ITEM-2
Vehicle
Anti-CSF1R

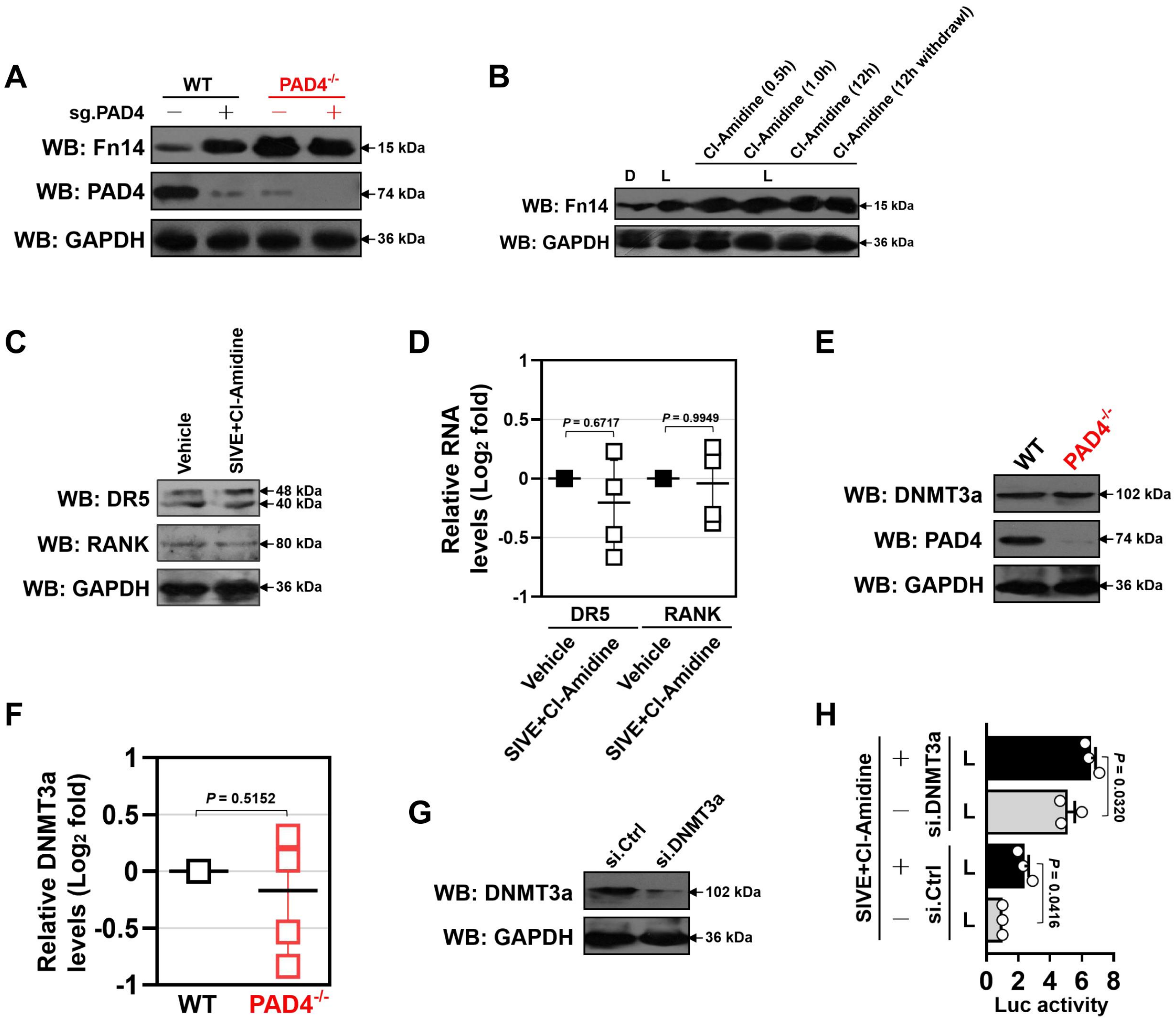
A
WT
PAD4-/-
sg.PAD4
WB: Fn14
WB: PAD4
WB: GAPDH
15 kDa
74 kDa
36 kDa
B
Cl-Amidine (0.5h)
Cl-Amidine (1.0h)
Cl-Amidine (12h)
Cl-Amidine (12h withdrawl)
D
L
L
WB: Fn14
WB: GAPDH
15 kDa
36 kDa
C
Vehicle
SIVE+Cl-Amidine
WB: DR5
WB: RANK
WB: GAPDH
48 kDa
40 kDa
80 kDa
36 kDa
D
Relative RNA levels (Log2 fold)
P = 0.6717
P = 0.9949
DR5
RANK
Vehicle
SIVE+Cl-Amidine
Vehicle
SIVE+Cl-Amidine
E
WT
PAD4-/-
WB: DNMT3a
WB: PAD4
WB: GAPDH
102 kDa
74 kDa
36 kDa
F
Relative DNMT3a levels (Log2 fold)
P = 0.5152
WT
PAD4-/-
G
si.Ctrl
si.DNMT3a
WB: DNMT3a
WB: GAPDH
102 kDa
36 kDa
H
SIVE+Cl-Amidine
si.DNMT3a
si.Ctrl
L
P = 0.0320
P = 0.0416
Luc activity

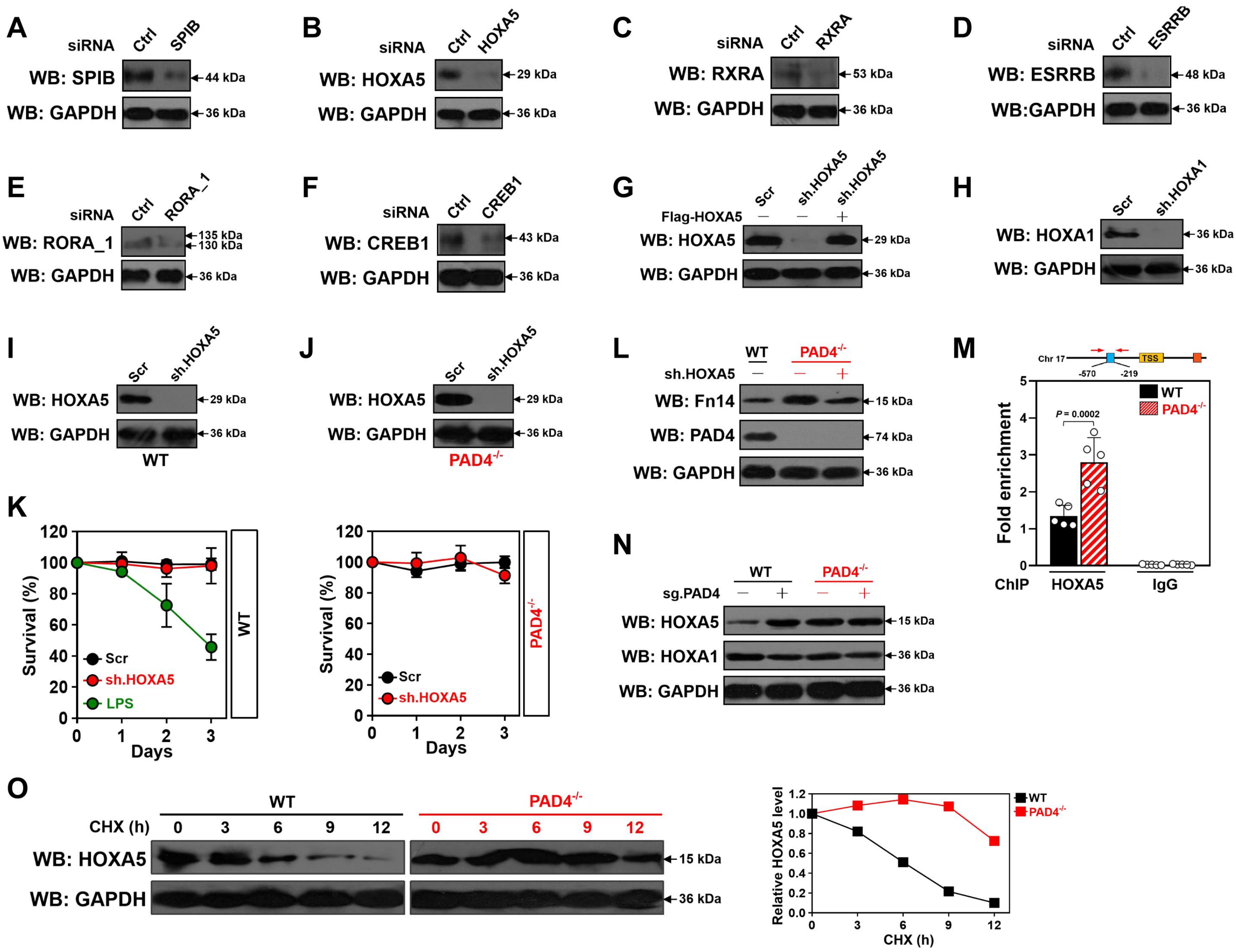
A
siRNA Ctrl SPIB
WB: SPIB 44 kDa
WB: GAPDH 36 kDa
B
siRNA Ctrl HOXA5
WB: HOXA5 29 kDa
WB: GAPDH 36 kDa
C
siRNA Ctrl RXRA
WB: RXRA 53 kDa
WB: GAPDH 36 kDa
D
siRNA Ctrl ESRRB
WB: ESRRB 48 kDa
WB:GAPDH 36 kDa
E
siRNA Ctrl RORA_1
WB: RORA_1 135 kDa 130 kDa
WB: GAPDH 36 kDa
F
siRNA Ctrl CREB1
WB: CREB1 43 kDa
WB: GAPDH 36 kDa
G
Scr sh.HOXA5 sh.HOXA5
Flag-HOXA5 − − +
WB: HOXA5 29 kDa
WB: GAPDH 36 kDa
H
Scr sh.HOXA1
WB: HOXA1 36 kDa
WB: GAPDH 36 kDa
I
Scr sh.HOXA5
WB: HOXA5 29 kDa
WB: GAPDH 36 kDa
WT
J
Scr sh.HOXA5
WB: HOXA5 29 kDa
WB: GAPDH 36 kDa
PAD4-/-
L
WT PAD4-/-
sh.HOXA5 − − +
WB: Fn14 15 kDa
WB: PAD4 74 kDa
WB: GAPDH 36 kDa
M
Chr 17 -570 -219 TSS
WT PAD4-/-
P = 0.0002
Fold enrichment
ChIP HOXA5 IgG
K
Survival (%)
Scr
sh.HOXA5
LPS
Days
WT
Survival (%)
Scr
sh.HOXA5
Days
PAD4-/-
N
WT PAD4-/-
sg.PAD4 − + − +
WB: HOXA5 15 kDa
WB: HOXA1 36 kDa
WB: GAPDH 36 kDa
O
WT PAD4-/-
CHX (h) 0 3 6 9 12 0 3 6 9 12
WB: HOXA5 15 kDa
WB: GAPDH 36 kDa
Relative HOXA5 level
WT
PAD4-/-
CHX (h)

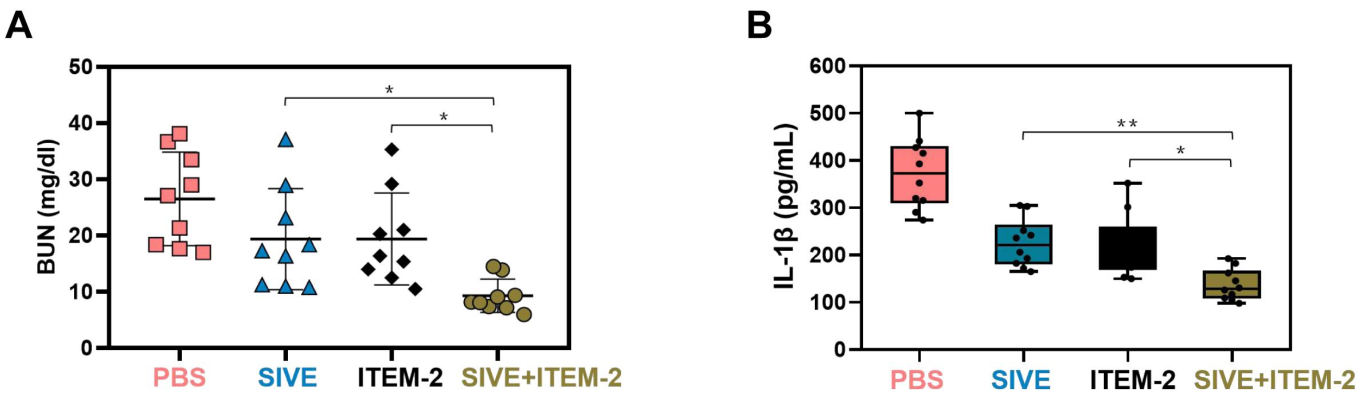
A
B
BUN (mg/dl)
50
40
30
20
10
0
*
*
PBS
SIVE
ITEM-2
SIVE+ITEM-2
IL-1β (pg/mL)
600
500
400
300
200
100
0
**
*
PBS
SIVE
ITEM-2
SIVE+ITEM-2

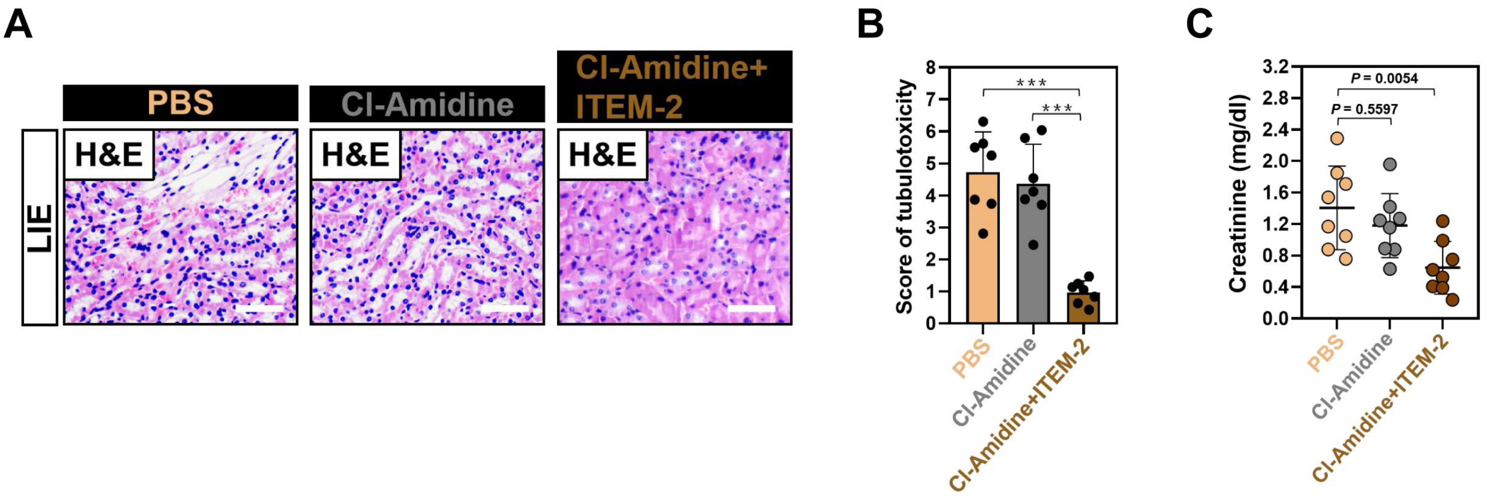

A
PBS
Cl-Amidine
Cl-Amidine+ ITEM-2
H&E
LIE
B
Score of tubulotoxicity
***
PBS
Cl-Amidine
Cl-Amidine+ITEM-2
C
Creatinine (mg/dl)
P = 0.0054
P = 0.5597
PBS
Cl-Amidine
Cl-Amidine+ITEM-2

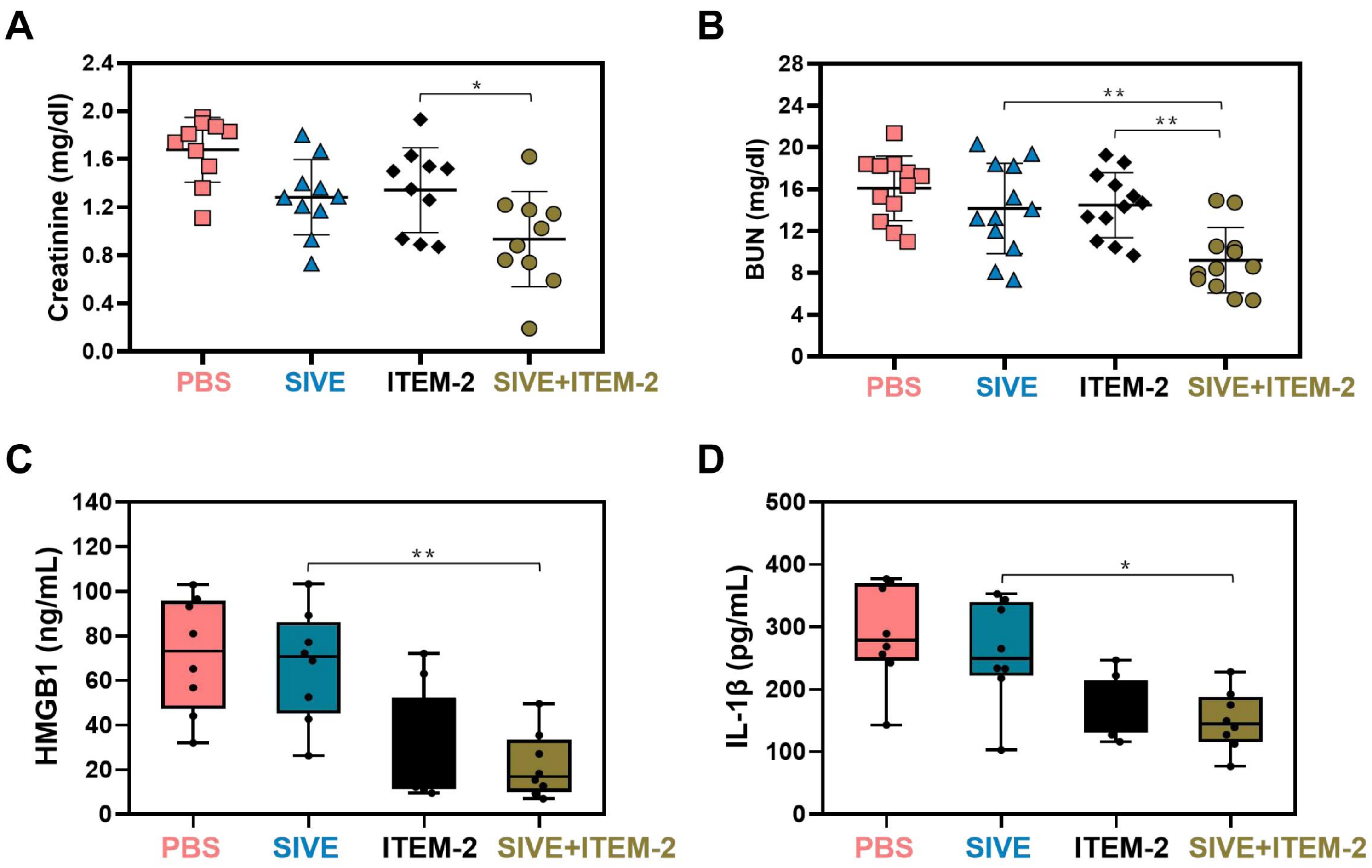

A
Creatinine (mg/dl)
2.4
2.0
1.6
1.2
0.8
0.4
0.0
*
PBS
SIVE
ITEM-2
SIVE+ITEM-2
B
BUN (mg/dl)
28
24
20
16
12
8
4
0
**
**
PBS
SIVE
ITEM-2
SIVE+ITEM-2
C
HMGB1 (ng/mL)
140
120
100
80
60
40
20
0
**
PBS
SIVE
ITEM-2
SIVE+ITEM-2
D
IL-1β (pg/mL)
500
400
300
200
100
0
*
PBS
SIVE
ITEM-2
SIVE+ITEM-2

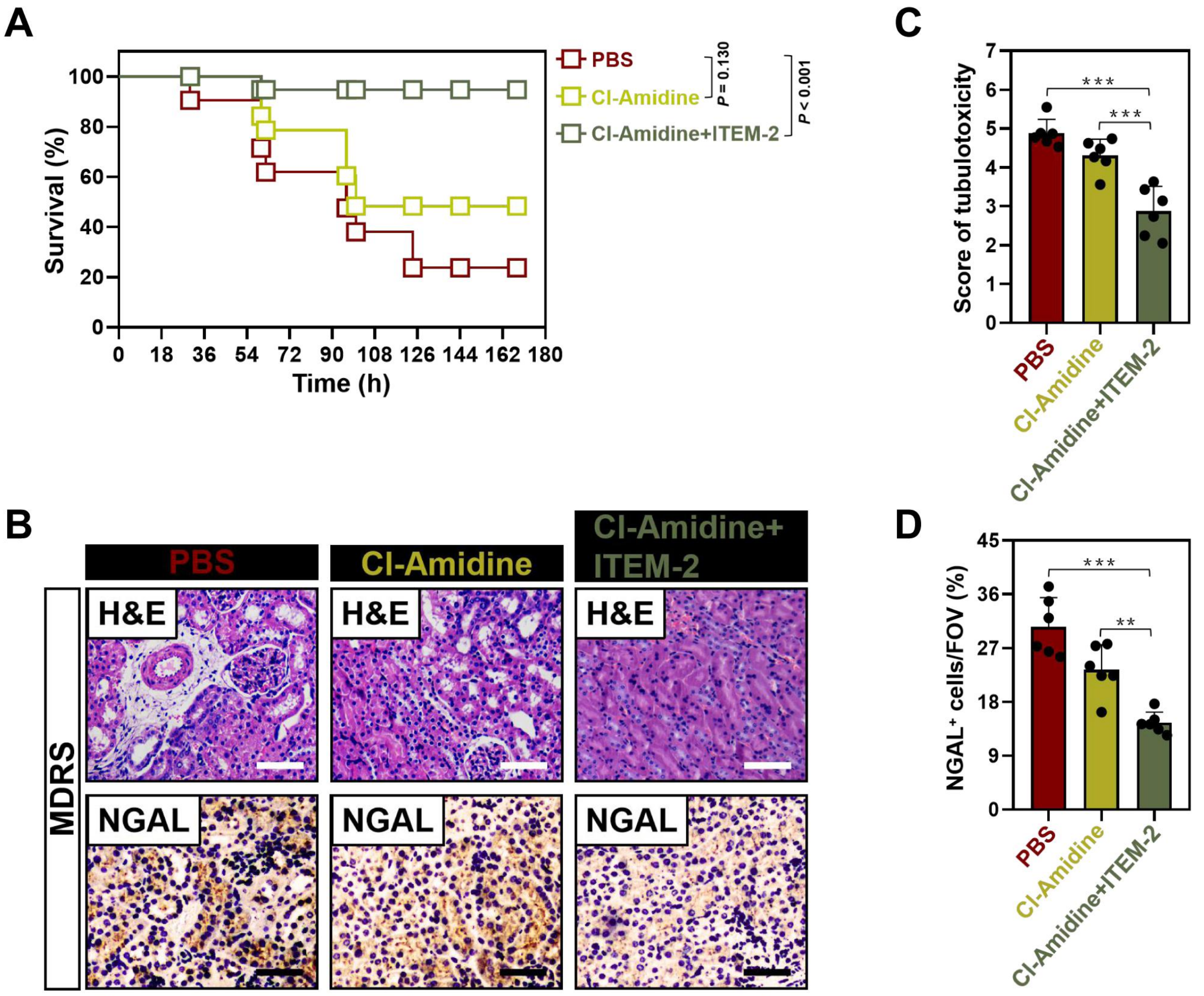

A
PBS
Cl-Amidine
Cl-Amidine+ITEM-2
P = 0.130
P < 0.001
Survival (%)
Time (h)
B
PBS
Cl-Amidine
Cl-Amidine+ITEM-2
MDRS
H&E
NGAL
C
Score of tubulotoxicity
***
***
D
NGAL⁺ cells/FOV (%)
***
**

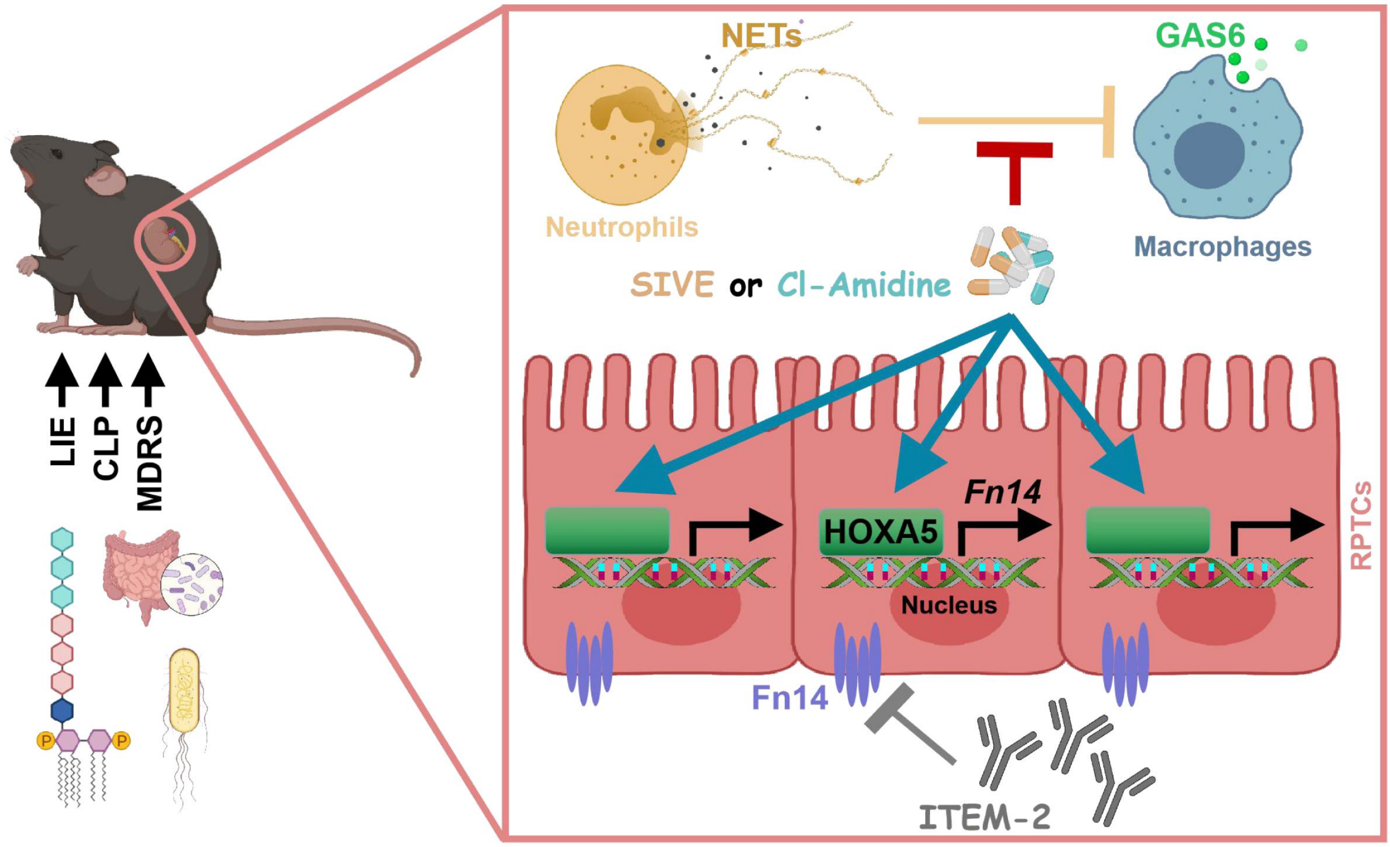

LIE
CLP
MDRS
NETs
GAS6
Neutrophils
Macrophages
SIVE or Cl-Amidine
Fn14
HOXA5
Nucleus
RPTCs
Fn14
ITEM-2

Table S1 Patient information for serum NETs and Fn14 measurement

Patients number	Age	Gender	SOFA score	KIDIGO stage	Patients number	Age	Gender	SOFA score	KIDIGO stage
1	67	Male	N/A	N/A	40	38	Female	N/A	N/A
2	67	Male	N/A	N/A	41	32	Male	N/A	N/A
3	66	Male	N/A	N/A	42	31	Female	N/A	N/A
4	65	Male	N/A	N/A	1	52	Female	24	III
5	65	Female	N/A	N/A	2	52	Female	24	III
6	63	Female	N/A	N/A	3	49	Male	21	III
7	61	Male	N/A	N/A	4	48	Male	18	III
8	61	Male	N/A	N/A	5	47	Male	17	III
9	61	Male	N/A	N/A	6	47	Male	16	III
10	60	Female	N/A	N/A	7	47	Male	16	III
11	60	Male	N/A	N/A	8	47	Male	15	III
12	59	Male	N/A	N/A	9	46	Male	15	III
13	59	Male	N/A	N/A	10	46	Male	14	III
14	56	Female	N/A	N/A	11	46	Female	13	III
15	55	Female	N/A	N/A	12	46	Male	12	III
16	54	Male	N/A	N/A	13	45	Female	11	III
17	53	Male	N/A	N/A	14	45	Female	11	III
18	53	Female	N/A	N/A	15	45	Female	11	III
19	53	Male	N/A	N/A	16	45	Female	10	III
20	53	Male	N/A	N/A	17	45	Male	10	III
21	52	Male	N/A	N/A	18	45	Male	9	III
22	52	Male	N/A	N/A	19	45	Female	8	III
23	51	Male	N/A	N/A	20	40	Male	8	III
24	51	Female	N/A	N/A	21	38	Male	7	III
25	51	Male	N/A	N/A	22	81	Male	17	II
26	50	Female	N/A	N/A	23	80	Male	16	II
27	49	Male	N/A	N/A	24	77	Male	15	II
28	48	Female	N/A	N/A	25	77	Male	14	II
29	46	Male	N/A	N/A	26	77	Male	14	II
30	45	Male	N/A	N/A	27	77	Female	13	II
31	44	Female	N/A	N/A	28	75	Female	12	II
32	43	Female	N/A	N/A	29	73	Male	12	II
33	43	Male	N/A	N/A	30	71	Male	10	II
34	42	Male	N/A	N/A	31	69	Male	10	II
35	42	Female	N/A	N/A	32	68	Female	10	II
36	42	Male	N/A	N/A	33	66	Male	9	II
37	42	Female	N/A	N/A	34	66	Male	9	II
38	41	Female	N/A	N/A	35	65	Male	9	II
39	40	Female	N/A	N/A	36	63	Female	9	II

37	62	Female	8	II	67	60	Female	4	I
38	61	Male	8	II	68	59	Female	4	I
39	60	Male	7	II	69	59	Male	4	I
40	60	Male	7	II	70	58	Male	4	I
41	60	Male	7	II	71	58	Male	3	I
42	60	Female	7	II	72	57	Male	3	I
43	59	Male	7	II	73	56	Female	3	I
44	56	Male	6	II	74	55	Male	3	I
45	56	Male	6	II	75	54	Male	3	I
46	54	Female	6	II	76	53	Male	3	I
47	53	Female	5	II	77	53	Female	2	I
48	52	Female	5	II	78	52	Female	2	I
49	52	Female	5	II	79	52	Male	2	I
50	51	Male	5	II	80	51	Male	2	I
51	51	Male	5	II	81	50	Male	2	I
52	50	Male	5	II	82	50	Male	2	I
53	46	Male	4	II	83	48	Male	2	I
54	46	Female	4	II	84	48	Male	2	I
55	43	Male	4	II	85	47	Male	2	I
56	43	Male	3	II	86	45	Male	2	I
57	35	Female	2	II	87	43	Male	2	I
58	75	Male	7	I	88	41	Male	2	I
59	68	Male	6	I	89	39	Male	2	I
60	66	Female	6	I	90	36	Male	2	I
61	63	Female	6	I	91	35	Male	2	I
62	62	Female	5	I	92	30	Male	2	I
63	61	Male	5	I	93	29	Male	2	I
64	60	Male	4	I					
65	60	Male	4	I					
66	60	Male	4	I					

Note: The plasma of 42 healthy volunteers and 93 patients clinically diagnosed with septic AKI were collected for neutrophilelastase (NE) and Fn14 measurement.
